# Could inflammation contribute to salivary gland dysfunction in patients with chronic heart failure?

**DOI:** 10.3389/fimmu.2022.1005981

**Published:** 2022-10-10

**Authors:** Anna Klimiuk, Anna Zalewska, Małgorzata Knapp, Anna Skutnik-Radziszewska, Mateusz Maciejczyk

**Affiliations:** ^1^ Experimental Dentistry Laboratory, Medical University of Bialystok, Bialystok, Poland; ^2^ Department of Cardiology, Medical University of Bialystok, Bialystok, Poland; ^3^ Department of Hygiene, Epidemiology and Ergonomics, Medical University of Bialystok, Bialystok, Poland

**Keywords:** chronic heart failure, salivary glands, hyposalivation, inflammation, saliva

## Abstract

Heart failure (HF) is one of the leading causes of death worldwide. HF results not only in cardiovascular dysfunction, but also numerous pathologies in the oral cavity and salivary glands. The present study is the first to evaluate whether salivary inflammatory and anti-inflammatory factors may be related with the occurrence of hyposalivation in HF patients. We also evaluated the potential of salivary biomarkers in the diagnostics of HF. The study included 30 women with HF and 30 sex- and age-matched healthy controls. We demonstrated significantly higher levels of pro-inflammatory cytokines, anti-inflammatory cytokines, Th1, Th2, Th17, chemokines and growth factors in unstimulated saliva of HF patients compared to controls. However, the results do not indicate dominance of either branch of the immune response. The concentration of selected biomarkers is significantly higher in patients with HF and salivary gland dysfunction compared to patients with normal saliva secretion and healthy subjects (IL-1β, TNF-α, IL-7, IL-13, INF-γ, IL-12, IL-15, IL-5, IL-6, IL-9, IL-17, MCP-1/CCL-2, EOTAXIN/CCL11, RANTES/CCL5, GM-CSF, VEGF, FGF basic, PDFG-BB). Multivariate regression analysis showed that the content of salivary cytokines, chemokines and growth factors is highly dependent on salivary gland function, i.e. salivary flow rate, total protein content and amylase activity. Using receiver operating characteristic (ROC) analysis, we showed that salivary TNF-α, INF-γ, IL-12 and EOTAXIN/CCL11 differentiated patients with HF and hyposalivation with the highest sensitivity and specificity compared to patients with normal salivary secretion and controls. Interestingly, the content of some pro- and anti-inflammatory mediators in saliva significantly exceeds their concentration in plasma. In addition, salivary biomarker levels do not reflect their plasma content, which may suggest a different nature/severity of inflammatory changes at the central (blood) and local (salivary) levels. Although our study was purely observational, the significantly higher concentration of inflammatory parameters in saliva compared to plasma, as well as the lack of saliva-blood correlation, may suggest increased production/secretion of these compounds in salivary cells of HF patients. ROC analysis did not confirm the diagnostic utility of salivary cytokines and chemokines in the differential diagnosis of HF patients.

## Introduction

Despite enormous medical advances, chronic heart failure (HF) remains a huge social and clinical problem. It is connected with continuously aging of the population and the resulting increased incidence of cardiovascular diseases (1). HF is the most common and the most expensive cause of hospitalization of people over 65 years of age and is connected with the highest risk of death. The risk of HF increases with age, from about 1% in people younger than 55 to over 10% in people who are over 70 years old (2–4). Heart failure occurs when the cardiac muscle loses its ability to pump blood efficiently because the ventricles cannot contract and relax properly, causing insufficient blood supply to strategically important organs. Although the basis of HF therapy is pharmacological treatment, it is a major clinical challenge in older patients. Indeed, comorbidities require the use of numerous additional drugs, which exacerbates the risk of interactions and side effects (5–8). Many of the side effects of cardiovascular medicines occur within the oral cavity, which significantly aggravates the symptoms of the underlying disease. These drugs include, among others, anticoagulants (9). Patients with HF present decreased saliva production (hyposalivation), impaired salivary protein secretion and a subjective sensation of dry mouth (xerostomia) (10). Hyposalivation is accompanied by difficulties in the formation and swallowing of food bites, impaired taste perception and problems with phonation. Other frequent symptoms related to salivary gland dysfunction include painful lesions in the tongue periphery, atrophy and dryness of the oral mucosa, and increased incidence of caries and periodontal disease, particularly in the cervical region of teeth (11–16). It is suggested that an inflammatory process may be responsible for salivary gland dysfunction. Previous studies have demonstrated a key role of oxidative stress in salivary gland secretory dysfunction in HF patients (17, 18). Indeed, patients with HF experience accumulation of protein, lipid and DNA oxidation products in the salivary-gland parenchyma, which impairs unstimulated and stimulated saliva production. In addition, activation of RAGE receptors (receptors for advanced glycation end products (AGEs)) can result in increased secretion of TNF-α (tumor necrosis factor alpha), IL-1 (interleukin-1) and IL-6 (interleukin-6), as well as the growth factors: IGF-1 (insulin-like growth factor 1) and TGF-β (transforming growth factor beta) which activate cells of the immune system. These conditions lead to overexpression of NADPH oxidase (NOX), which is a source of not only reactive oxygen species (ROS) but also numerous pro-inflammatory factors (19, 20). It is assumed that assessment of the inflammatory profile in saliva and gingival crevicular fluid (GCF) may have great diagnostic potential in terms of early detection of oral and systemic diseases (21, 22). Recent studies indicate that salivary endothelin may be a local indicator of inflammation in patients with coronary heart disease and periodontal disease (15). However, the levels of cytokines, chemokines and growth factors in the saliva of HF patients have yet to be determined.

This study is the first to evaluate whether salivary inflammatory and anti-inflammatory factors may be connected with the occurrence of hyposalivation in HF patients. Subjects qualified for the study were patients with HF who had undergone a comprehensive dental and sialometric examination, and then divided into groups with normal and reduced saliva secretion. Thus, our study may contribute to better understanding of the causes of salivary gland dysfunction that significantly impairs the quality of life of HF patients. In the future, it may contribute to the development of new therapeutic strategies for hyposalivation treatment in patients with HF. Bearing in mind that salivary biomarkers are used in the diagnosis of a number of systemic diseases, an additional aim of our study was to determine whether the assessment of levels of salivary cytokines, chemokines and growth factors may have diagnostic value in patients with HF.

## Materials and methods

### Ethical issues

The study was approved by the Bioethics Committee of the Medical University of Bialystok, Poland (permission number R-I-002/75/2016). All subjects gave their written consent to participate in the experiment after a thorough explanation of the purpose of the study and possible risks related to it.

### Patients

The criteria for inclusion and exclusion from the study are presented in **Table 1**. It should be noted that subjects with systemic, autoimmune and oral diseases were eliminated from the study, allowing an objective assessment of the salivary inflammatory profile.

**Table 1:** Inclusion and exclusion criteria of HF patients and the control group.

	Inclusion criteria	Exclusion criteria
**Control and study group**	- written informed consent to participate in the study - age 55–90 - BMI 18.5–24.5 - normal results of blood morphology (WBC, RBS, HGB, HCT, PLT) and biochemical tests (CRP, GFR, TSH, AST, glucose)	- presence of chronic systemic and autoimmune diseases (type 1 diabetes, Sjogren’s syndrome, rheumatoid arthritis, psoriasis) or lung, thyroid, liver, kidney, gastrointestinal diseases, infectious diseases (HCV, HBV and HIV infection) and immune system disorders - periodontal diseases - oral cancer - smoking - drinking alcohol - taking antibiotics, non-steroidal anti-inflammatory drugs (NSAIDs), glucocorticoids, dietary supplements and vitamins for the last 3 months
**Study group**	- chronic hemodynamically stable heart failure (class II (NYHA II) and III (NYHA III) failure according to the New York Heart Association) - left ventricular ejection fraction (LVEF) <35% - patients qualified for implantation of an automatic cardioverter-defibrillator or cardiac resynchronization therapy system	- chronic hemodynamically unstable heart failure
**Control group**	- normal cardiac performance – left ventricular ejection fraction (LVEF) >55%	- chronic heart failure

The study group consisted of 30 women with chronic heart failure, treated at the Department of Cardiology with the Intensive Cardiac Care Unit of the Medical University of Bialystok Clinical Hospital in Bialystok. All patients were qualified for the study by an experienced cardiologist (M. K.) based on the inclusion and exclusion criteria. Material for the study was collected before implantation of an automatic cardioverter-defibrillator or a cardiac resynchronization system.

The control group, matched in terms of gender and age to the study group, consisted of 30 generally healthy patients attending follow-up appointments at the Outpatient Clinic for Restorative Dentistry of the Specialized Dental Department of the Medical University of Bialystok.

### Saliva collection

The study material consisted of unstimulated whole saliva collected from patients *via* the spitting method. In order to minimize the effect of diurnal rhythm on saliva secretion, samples were collected in the morning, between 7 and 9 a.m., under conditions that eliminated stimuli. On the day of the study, prior to saliva collection, subjects from the study/control group did not have any meals (last meal at least 10 hours earlier) or beverages (excluding clean water) and refrained from performing oral hygiene procedures. They also had not taken any medications at least 8 hours before saliva collection. After rinsing the mouth twice with room-temperature distilled water, patients spat out saliva accumulated at the bottom of the mouth, for 15 minutes, into a sterile centrifuge tube placed in a container with ice (23). The volume of saliva was measured with a single-channel pipette with accuracy of 100 μL and was the basis for calculating the salivary flow rate (SFR; mL/min). The collected saliva was immediately centrifuged (3000 x g, 20 minutes, +4°C). Butylated hydroxytoluene (BHT) (5 μl of 0.5 M BHT in acetonitrile per 0.5 mL of salivary supernatant fluid) was added to the obtained supernatants to protect them against oxidation processes. The samples were stored at -80°C for no longer than six months.

### Sialochemistry

Hyposalivation was defined as saliva flow rate (SFR) below 0.2 mL/min (23–26). Based on SFR, patients with HF were divided into two groups: a group with normal salivation (NS) and one with reduced saliva secretion (hyposalivation, HS). All control patients had SFR above 0.2 mL/min.

In addition to SFR, total protein (TP) content and salivary amylase activity were assessed to evaluate salivary gland function. TP content was determined colorimetrically with a commercial Thermo Scientific PIERCE BCA Protein Assay kit (Rockford, IL, USA) using the bicinchoninic method. Bicinchoninic acid (BCA) forms a stable complex with copper (2+) ions, which demonstrates an absorption maximum at 562 nm wavelength. Total protein concentration was expressed in μg/mL. Salivary amylase (SA, EC 3.2.1.1) activity was determined colorimetrically at 540 nm wavelength using 3’,5’-dinitrosalicylic acid (DNS). Absorbance changes accompanying the increase in the concentration of reducing sugars released during starch hydrolysis catalyzed by salivary amylase were also measured (27, 28). Salivary amylase activity was determined in duplicate samples and expressed in μg/mg of total protein.

### Dental examination

The dental examination of every patient was performed immediately after saliva collection by the same dentist (A. K.), according to the criteria of the World Health Organization – in artificial lighting, using a mirror, an explorer and a periodontal probe (29). DMFT (decayed, missing, filled teeth), PBI (Papilla Bleeding Index), GI (Gingival Index) were determined. The DMFT index is the sum of teeth with caries (D), teeth extracted because of caries (M), and teeth filled because of caries (F). The PBI showed the intensity of bleeding from the gingival papilla after probing (30, 31). Criteria connected with GI included qualitative changes in the gingiva (32). Moreover, inter-rater agreements were assessed in 15 patients. The reliability coefficient for DMFT was r = 0.95; for PBI it was r = 0.96; and for GI: r = 0.97.

### Blood collection

Venous blood (10 ml) was collected from subjects upon fasting and after an overnight rest, using the S-Monovette^®^ K3 EDTA blood collection system (Sarstedt). Blood samples were centrifuged (1500 x g, 10 minutes, +4°C; MPW 351, MPW Med. Instruments, Warsaw, Poland), and plasma was collected for assays. The antioxidant butylated hydroxytoluene (BHT) was added to the samples (23). Samples were stored at -80°C for no longer than six months.

### Bio-plex multiplex assay

The concentration of salivary and plasma cytokines: inflammatory (IL-1β: interleukin 1β; TNF-α: tumor necrosis factor α; IL-7: interleukin 7), anti-inflammatory (IL-10: interleukin 10; IL-1ra: interleukin 1RA; IL-13: interleukin 13), Th1 (INF-γ: interferon γ; IL-12: interleukin 12; IL-2: interleukin 2; IL-15: interleukin 15), Th2 (IL-4: interleukin 4; IL-5: interleukin 5; IL-6: interleukin 6; IL-9: interleukin 9) and Th17 (IL-17: interleukin 17), as well as chemokines (IP-10/CXCL10: chemokine (C-X-C motif) ligand 10/interferon gamma-induced protein 10; MCP-1/CCL2: monocyte chemoattractant protein-1; MIP-1α/CCL3: chemokine ligands 3/macrophage inflammatory protein 1α; MIP-1β/CCL4: chemokine ligands 4/macrophage inflammatory protein 1β; CCL11/eotaxin: chemokine ligand 11/eotaxin; CCL5/RANTES: chemokine ligand 5/regulated on activation, normal T cell expressed and secreted; IL-8/CXCL8: interleukin 8) and growth factors (G-CSF: granulocyte colony-stimulating factor; GM-CSF: granulocyte-macrophage colony-stimulating factor; VEGF: vascular endothelial growth factor; FGF basic: fibroblast growth factor; PDFG-BB: platelet-derived growth factor) were determined using the Bio-Plex Pro Human Cytokine 27-plex Assay commercial diagnostic kit (Bio-Rad Laboratories, Inc., Hercules, CA, USA). Bio-Plex technology is a multiplex-type ELISA test based on magnetic beads. Captured antibodies directed against a specific biomarker are covalently bound to magnetic beads. The coupled beads then react with a sample containing the selected biomarker. A series of rinses is performed to remove the unbound protein, and then a biotinylated detection antibody is added to form a sandwich compound. The final complex is formed by adding streptavidin-phycoerythrin (SA-PE) conjugate. The reading is performed using a specialized plate reader (Bio-Plex 200), and this method’s performance can be compared to a typical ELISA test.

### Statistical analysis

Statistical analysis of the results was performed using GraphPad Prism 8.4.3. for MacOS (GraphPad Software, La Jolla, USA). The D’Agostino-Pearson test and Shapiro-Wilk test were used to assess normality of distribution, and homogeneity of variance was evaluated by means of the Levene’s test. The results are presented in box-and-whisker plots as median (minimum-maximum). For comparisons between the two groups, the Whitney U-Mann test was used. Kruskal-Wallis ANOVA analysis of variance along with Dunn’s *post hoc* test was applied for comparisons between the multiple groups. Correlations between biomarkers and salivary gland secretory function were assessed using Spearman’s rho correlation coefficient.

Multivariate analysis of simultaneous impact of many independent variables (SFR, TP, SA) on one quantitative dependent variable was performed by means of linear regression. Confidence intervals of 95% were reported along with regression parameters. Analysis of the diagnostic utility of biomarkers was measured using the receiver operating characteristic (ROC) analysis. A p value of less than 0.05 was considered statistically significant.

The number of patients was determined *a priori* based on the previously conducted pilot study, and the power of the test was assumed to be 0.9. Salivary TNF-α, IL-13, INF-γ and MCP-1/CCL-2 were used for the calculation. The minimum number of patients in one group was 21; thus, 30 patients were qualified for the study.

## Results

### Clinical data

The clinical data of the patients are presented in **Table 2**. In general, HF patients with hyposalivation or normal saliva secretion did not differ from the control group in terms of blood count, biochemical tests, RR, comorbidities or medications taken.

**Table 2:** Clinical characteristics of HF patients and the control group.

Paremeter	Control group n=30	Study group n=30	Study NS n=17	Study HS n=13	P-value			
					ANOVA	Study NS vs. Control	Study HS vs. Control	Study HS vs. Study NS
Age	63.23 ± 9.27	63.23 ± 9.27	65.47 ± 9.53	60.31 ± 8.37	0.5116	0.8536	0.7734	0.4278
Blood morphology								
WBC (x 10^3^/µL)	7.52 ± 0.39	7.3 ± 1.9	6.98 ± 1.71	7,63 ± 2.1	0.6629	0.7079	0.9963	0.6972
RBC (x 10^6^/µL)	4.49 ± 0.35	4.34 ± 0.58	4.36 ± 0.69	4.32 ± 0.47	0.6528	0.87	0.7538	0.9965
HGB (g/dL)	13.64 ± 2.31	13.13 ± 1.38	13.10 ± 1.31	13.16 ± 1.49	0.6654	0.7871	0.8516	0.9997
HCT (%)	39.08 ± 2.72	38.69 ± 3.91	38.38 ± 3.89	39.03 ± 4.05	0.9269	0.9274	>0.9999	0.9638
PLT (x10^3^/µL)	243.3 ± 78.67	183.6 ± 44.97	178.6 ± 50.38	189.1 ± 39.62	**0.0005**	**0.0065**	**0.0373**	0.9679
Biochemical tests								
CRP (mg/L)	2.93 ± 0.24	4.53 ± 10.35	3.03 ± 1.95	2.53 ± 2.74	0.713	>0.9999	0.9974	0.997
GFR (ml/min)	86.99 ± 5.78	78.67 ± 20.45	80.21 ± 17.38	77 ± 23.94	0.1832	0.6006	0.2865	0.9598
TSH (µIU/mL)	1.04 ± 0.2	1.218 ± 1.04	0.82 ± 0.5	1.56 ± 1.26	0.1233	0.8543	0.2237	0.1185
AST (IU/L)	21.52 ± 4.54	23.5 ± 7.04	23.8 ± 7.09	23.33 ± 7.17	0.5728	0.6413	0.9103	0.9989
Glucose (mg/dL)	90.24 ± 4.35	96.25 ± 7.97	94.89 ± 7.46	97.36 ± 8.56	**0.0074**	0.2732	**0.0192**	0.8434
RR								
SBP (mmHg)	125.2 ± 2.35	128.0 ± 20.06	129.4 ± 23.24	126.6 ± 16.81	0.8509	0.8558	0.9933	0.9715
DBP (mmHg)	70.44 ± 3.95	76.15 ± 11.83	74.64 ± 13.75	77.77 ± 9.64	0.0723	0.5482	0.1172	0.84
Systemic diseases								
Type 2 diabetes *n* (%)	12 (40)	12 (40)	7 (41.18)	5 (38.46)	NA			
Hypertension *n* (%)	24 (80)	24 (80)	12 (70.59)	12 (92.31)	NA			
Coronary artery disease *n* (%)	0 (0)	7 (23.33)	4 (23.53)	3 (23.08)	NA			
Myocardial infarction *n* (%)	0 (0)	2 (6.67)	1 (5.88)	1 (7.69)	NA			
Pharmacotherapy								
ACE *n* (%)	4 (13.33)	9 (30)	4 (23.53)	5 (38.46)	NA			
Alpha receptor blocker *n* (%)	0 (0)	3 (10)	1 (5.88))	2 (15.38)	NA			
AT1 receptor blocker *n* (%)	1 (3.33)	3 (10)	2 (11.76)	1 (7.69)	NA			
ASA *n* (%)	6 (20)	9 (30)	5 (29.41)	4 (30.77)	NA			
Beta receptor blocker *n* (%)	7 (23.33)	11 (36.67)	5 (29.41)	6 (46.15)	NA			
Ca^2+^ channel blocker *n* (%)	3 (10)	4 (13.33)	2 (11.76)	2 (15.38)	NA			
Cardiac glycosides *n* (%)	0 (0)	2 (6.67)	1 (5.88)	1 (7.69)	NA			
Diuretics *n* (%)	2 (6.67)	3 (10)	2 (11.76)	1 (7.69)	NA			
OADs *n* (%)	12 (40)	12 (40)	7 (41.18)	5 (38.46)	NA			
Organic nitrate *n* (%)	0 (0)	1 (3.33)	0 (0)	1 (7.69)	NA			
Statins *n* (%)	8 (26.67)	13 (43.33)	7 (41.18)	6 (46.15)	NA			

Bold indicates statistically significant results. NA, non applicable.

### Sialochemistry and dental examination

The results of the sialochemistry and dental examination are presented in **Table 3**. We observed significantly lower SFR in HF patients with HS compared to controls and HF patients with normal salivary secretion (in all cases p < 0.001). In addition to SFR, total protein content and salivary amylase activity were assessed to evaluate salivary gland function. Total protein content and salivary amylase activity were significantly lower in the saliva of HS patients compared to controls (p = 0.0296, p = 0.0003, respectively). However, patients in the study group and the control did not differ in terms of oral hygiene, caries and periodontal status (**Table 3**).

**Table 3:** Sialochemistry and dental examination of HF patients and control subjects.

Parameter	Control group n=30	Study group n=30	Study NS n=17	Study HS n=13	P-value			
					ANOVA	Study NS vs. Control	Study HS vs. Control	Study HS vs. Study NS
Salivary gland function								
SFR (mL/min)	0.38 ± 0.05	0.29 ± 0.12	0.37 ± 0.09	0.18 ± 0.03	**<0.0001**	>0.9999	**<0.0001**	**<0.0001**
TP (µg/mL)	1923 ± 589.7	1563 ± 553.2	1715 ± 549.7	1364 ± 511.2	**0.0204**	>0.9999	**0.0296**	0.694
SA (µmol/mg protein)	0.20 ± 0.07	0.14 ± 0.04	0.15± 0.02	0.14± 0.05	**<0.0001**	0.0705	**0.0003**	0.6414
Dental examination								
DMFT	17.63 ± 1,52	17.80 ± 1.5	17.53 ± 1.7	18.15 ± 1.14	0.6795	>0.9999	>0.9999	>0.9999
PBI	1.12 ± 0.33	1.11 ± 0.3	1.16 ± 0.22	1.05 ± 0.4	0.8755	>0.9999	>0.9999	>0.9999
GI	1.01 ± 0.61	1.01 ± 0.59	1.11 ± 0.59	0.88± 0.58	0.5722	>0.9999	>0.9999	0.951

DMFT, decayed, missing, filled teeth index; GI, gingival index; HS, hyposalivation; NS, normal salivation; PBI, papilla bleeding index; SA, salivary amylase; SFR, saliva flow rate; TP; total protein content. Bold indicates statistically significant results.

### Salivary inflammatory and anti-inflammatory profile in patients with heart failure compared to the controls

#### Pro-inflammatory cytokines

**Figure 1:**
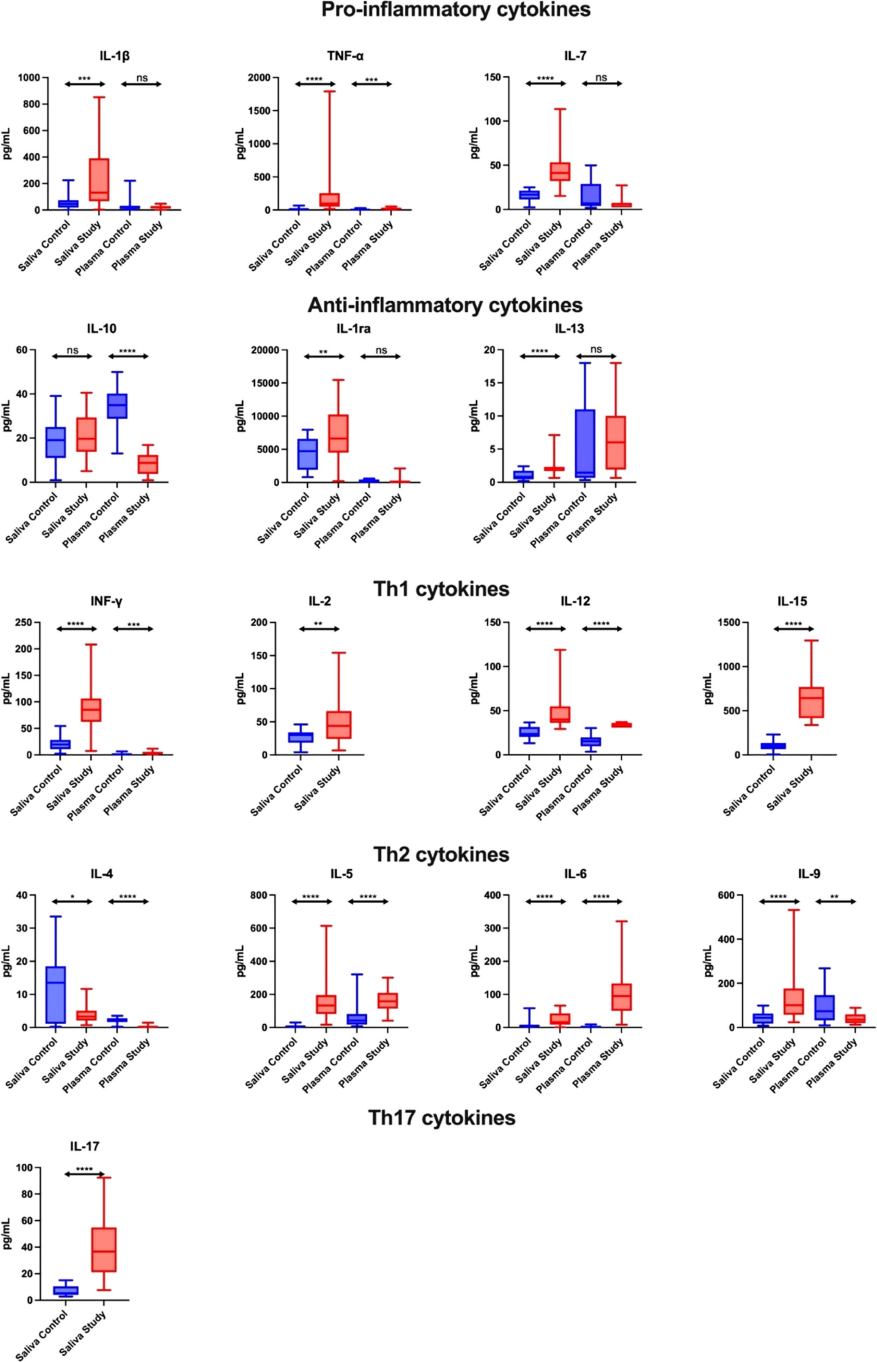
Salivary pro-inflammatory and anti-inflammatory profile in patients with heart failure (n = 30) compared to the control (n = 30). The results are presented in box-and-whisker plots as median (minimum-maximum). For comparisons between the two groups, the Whitney U-Mann test was used. *p< 0.05, **p< 0.01, ***p< 0.001, and ****p< 0.0001. NS, non significance.

The concentration of pro-inflammatory cytokines (IL-1β, TNF-α, IL-7) in the saliva of study group subjects was significantly higher compared to the control group (↑189%, p = 0.0002; ↑ 606%, p < 0.0001; ↑145%, p < 0.0001, respectively). A similar trend in plasma was observed only for TNF-α levels in the plasma of patients from the study group (↑141%, p = 0.0003). In addition, the concentration of IL-1β and TNF-α in saliva correlated positively with their plasma levels in both the control and study groups (**Figure 1**; **Table S1**).

#### Anti-inflammatory cytokines

The concentration of anti-inflammatory cytokines (IL-1ra, IL-13) in the saliva of study group subjects was statistically significantly higher compared to the control group (↑40%, p = 0.0081; ↑163%, p < 0.0001, respectively). In contrast, the serum level of IL-10 in the study group was significantly lower compared to the controls (↓ 75%, p < 0.0001). Moreover, the concentration of IL-10 and IL-1ra in the saliva of control subjects correlated positively with plasma levels of these cytokines (**Figure 1**; **Table S1**).

#### Th1 cytokines

The concentration of Th1 cytokines (INF-γ, IL-12, IL-2, IL-15) in the saliva of study group subjects was significantly higher than in the control group (↑332, p < 0.0001; ↑70%, p < 0.0001; ↑46%, p = 0.007; ↑531, p < 0.0001, respectively), while in plasma only INF-γ and IL-12 concentrations were higher in the study group compared to the controls (↑121%, p = 0.0003; ↑121, p < 0.0001, respectively). In addition, salivary INF-γ level correlated negatively with its plasma content in patients from the study group (**Figure 1**; **Table S1**).

#### Th2 cytokines

The content of Th2 cytokines in the case of determinations of IL-5, IL-6, IL-9 in the saliva of study group patients was significantly higher compared to the control group (↑1920%, p < 0.0001; ↑195%, p < 0.0001; ↑131%, p < 0.0001, respectively), while the concentration of IL-4 was significantly lower (↓76%, p = 0.0202). In terms of plasma determinations, only the levels of IL-5 and IL-6 were considerably higher in the study group than in the control group (↑286%, p < 0.0001; ↑4004%, p < 0.0001, respectively), while IL-4 and IL-9 concentrations were significantly lower (↓91%, p < 0.0001; ↓51%, p = 0.0022, respectively). In the control group, the salivary content of IL-4, IL-6 and IL-9 correlated positively with their plasma level, while the concentration of IL-5 correlated negatively. Moreover, IL-5 level in saliva was positively correlated with its plasma content in the study group (**Figure 1**; **Table S1**).

#### Th17 cytokines

The concentration of Th17 cytokines in the saliva of study group patients was significantly higher compared to the control group (↑591%, p < 0.0001) (**Figure 1**; **Table S1**).

#### Chemokines

**Figure 2:**
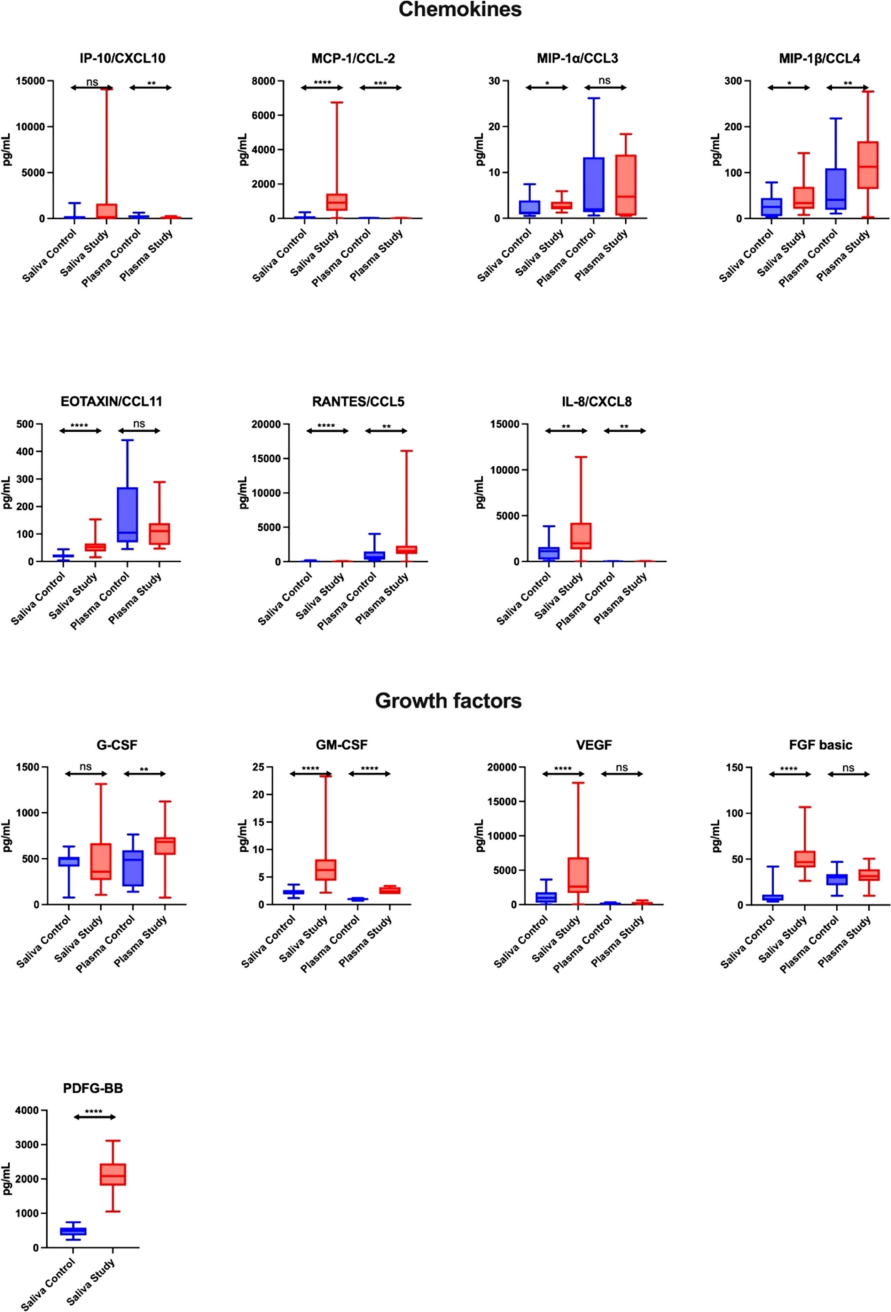
Salivary chemokines and growth factors in patients with heart failure (n = 30) compared to the control (n = 30). The results are presented in box-and-whisker plots as median (minimum-maximum). For comparisons between the two groups, the Whitney U-Mann test was used. *p< 0.05, **p< 0.01, ***p< 0.001, and ****p< 0.0001. NS, non significance.

The concentration of chemokines (MCP-1/CCL-2, IP-1α/CCL3, MIP-1β/CCL4, EOTAXIN/CCL11, RANTES/CCL5, IL-8/CXCL8) in the saliva of study group subjects was significantly higher compared to the control group (↑1329%, p < 0.0001; ↑100%, p = 0.0209; ↑33%, p = 0.0238; ↑159%, p < 0.0001; ↑477%, p < 0.0001; ↑76%, p = 0.0068, respectively), while in plasma only the levels of MIP-1β/CCL4 and RANTES/CCL5 (↑178%, p = 0.0022 and ↑139%, p = 0.0016, respectively) were higher in the study group vs. the controls. In the plasma of patients from the study group, the content of IP-10/CXCL10, MCP-1/CCL-2 and IL-8/CXCL8 was significantly lower compared to the control group (↓57%, p = 0.0015; ↓61%, p = 0.0003; ↓63%, p = 0.0025, respectively). In the control group, salivary concentration of MIP-1β/CCL4 and IL-8/CXCL8 correlated positively with their plasma levels, while the level of RANTES/CCL5 correlated negatively (**Figure 2**; **Table S1**).

#### Growth factors

The concentration of growth factors (GM-CSF, VEGF, FGF basic, PDFG-BB) in the saliva of study group patients was significantly higher compared to the control group (↑200%, ↑170%, ↑687%, ↑325%, respectively, p < 0.0001 in all cases), while in plasma only the levels of G-CSF and GM-CSF (↑40%, p = 0.0061; ↑140%, p < 0.0001, respectively) were higher in the study group vs. the controls. In the control group, salivary G-CSF and VEGF levels correlated positively with their plasma levels (**Figure 2**; **Table S1**).

### Inflammatory and anti-inflammatory profile in the saliva of patients with heart failure and hyposalivation compared to those with heart failure and normal saliva secretion and controls

#### Pro-inflammatory cytokines

The levels of TNF-α and IL-7 were significantly higher in the saliva of patients with heart failure and normal saliva secretion compared to the controls, as was the content of IL-1β, TNF-α and IL-7 in the saliva of patients in the study group with hyposalivation compared to the control group (**Table S2**).

#### Anti-inflammatory cytokines

Concentrations of IL-1ra, IL-13 were statistically significantly higher in the saliva of subjects with hyposalivation, and HF compared to the control group, while in the study group with HS, such a dependency was observed only for IL-13 level (**Table S2**).

#### Th1 cytokines

The levels of INF-γ, IL-12 and IL-15 were significantly higher in the saliva of heart failure subjects with both NS and HS compared to the control group (**Table S2**).

#### Th2 cytokines

The concentrations of IL-5, IL-6 and IL-9 were significantly higher in the saliva of heart failure patients with both NS and HS compared to the control group, while IL-4 level was significantly higher only in heart failure patients with HS (**Table S2**).

#### Th17 cytokines

Moreover, IL-17 level was significantly higher in the saliva of heart failure patients with both NS and HS compared to the controls (**Table S2**).

#### Chemokines

Concentrations of MCP-1/CCL-2, EOTAXIN/CCL11 and RANTES/CCL5 were significantly higher in the saliva of heart failure subjects with both NS and HS compared to the controls, while IL-8/CXCL8 level was considerably higher only in heart failure patients with NS (**Table S2**).

#### Growth factors

The levels of GM-CSF, VEGF, FGF basic and PDFG-BB were significantly higher in the saliva of heart failure patients with both NS and HS compared to the controls (**Table S2**).

### Correlations

#### Pro-inflammatory cytokines

Salivary concentrations of IL-1β and TNF-α were negatively correlated with salivary minute flow, protein content and salivary amylase activity in subjects from the study group; no such correlations were found in the control group (**Table S3**).

#### Th1 cytokines

The concentration of INF-γ and IL-12 correlated negatively with salivary minute flow, protein content and salivary amylase activity in the study group patients, while IL-12 content was additionally correlated negatively with salivary amylase activity in control subjects (**Table S3**).

#### Th2 cytokines

The concentration of IL-6 correlated positively with protein content in subjects in the study group. In contrast, IL-4 level correlated negatively with salivary amylase activity in the control subjects (**Table S3**).

#### Growth factors

The concentration of FGF basic was positively correlated with salivary amylase activity in control subjects (**Table S3**).

#### Regression analysis

Multivariate linear regression analysis enabled us to determine whether the content of salivary cytokines, chemokines and growth factors depend on the secretory activity of salivary glands. It was demonstrated that salivary IL-1β and TNF-α concentrations depend inversely on the minute flow, salivary protein content and salivary amylase activity, while INF-γ and IL-15 concentrations depend negatively on SFR and SA. The content of salivary IL-7 depends inversely on SA, while IL-5 depends on SFR, and PDFG-BB depends on SA (**Table 4**).

**Table 4:** Multivariate linear regression analysis. Salivary flow rate (SFR), total protein (TP) and salivary amylase (SA) were used as independent variables.

Biomarker	β1 (SFR)			β2 (TP)			β3 (SA)		
	Estimate	95% CI	P-value	Estimate	95% CI	P-value	Estimate	95% CI	P-value
Pro-inflammatory cytokines									
IL-1β	-1004	-1425 to -582.1	**<0.0001**	-0.14	-0.2242 to -0.05079	**0.0025**	-1260.00	-1990 to -528.8	**0.0011**
TNF-α	-1420	-2152 to -688.6	**0.0003**	-0.16	-0.3063 to -0.01220	**0.0344**	-1511.00	-2693 to -329.1	**0.0134**
IL-7	-48.99	-114.8 to 16.83	0.1411	0.01	-0.006534 to 0.01769	0.3593	-138.00	-236.5 to -39.45	**0.0070**
Anti-inflammatory cytokines									
IL-10	-28.58	-63.14 to 5.977	0.1027	0.01	-0.0007415 to 0.01136	0.0839	34.61	-15.06 to 84.29	0.1673
IL-1ra	678.9	-11588 to 12946	0.9118	1.44	-0.7203 to 3.606	0.1860	-10361.00	-27250 to 6528	0.2232
IL-13	-1.4	-5.164 to 2.364	0.4584	0.00002	-0.0006713 to 0.0007138	0.9510	-7.53	-13.17 to -1.899	**0.0098**
Th1 cytokines									
INF-γ	-203.2	-293.3 to -113.1	**<0.0001**	-0.01	-0.02559 to 0.009516	0.3621	-268.10	-412.4 to -123.8	**0.0005**
IL-12	-95.17	-149.8 to -40.57	**0.0010**	0.0001	-0.01158 to 0.01181	0.9839	-111.20	-231.0 to 8.631	0.0681
IL-2	-51.75	-168.5 to 65.02	0.3764	0.01	-0.01105 to 0.02759	0.3926	-101.20	-256.2 to 53.74	0.1947
IL-15	-1646	-2743 to -549.1	**0.0042**	0.09	-0.08248 to 0.2678	0.2915	-1437.00	-2738 to -136.9	**0.0311**
Th2 cytokines									
IL-4	12.40	-15.44 to 40.23	0.3749	0.0008	-0.004335 to 0.005998	0.7476	-0.66	-42.46 to 41.15	0.9749
IL-5	-364.70	-717.2 to -12.31	**0.0428**	0.08	0.01411 to 0.1405	**0.0177**	-321.70	-828.8 to 185.5	0.2078
IL-6	-16.06	-75.43 to 43.31	0.5890	0.01	-0.001201 to 0.01928	0.0823	-53.39	-136.3 to 29.56	0.2018
IL-9	-135	-413.8 to 143.9	0.3353	0.04	-0.01059 to 0.09260	0.1166	-369.60	-785.9 to 46.67	0.0805
Th17 cytokines									
IL-17	-71.61	-165.6 to 22.42	0.1305	0.01	-0.008725 to 0.02322	0.3619	-114.50	-244.0 to 15.01	0.0811
Chemokines									
IP-10/CXCL10	461.1	-11789 to 12711	0.9395	0.53	-1.737 to 2.796	0.6378	-5490.00	-23305 to 12326	0.5353
MCP-1/CCL-2	966.5	-4713 to 6646	0.7316	0.91	-0.04701 to 1.874	0.0616	-3751.00	-11428 to 3927	0.3278
MIP-1α/CCL3	1.73	-5.336 to 8.794	0.6237	0.0003	-0.0009817 to 0.001500	0.6756	-1.04	-10.85 to 8.772	0.8318
MIP-1β/CCL4	-76.88	-210.4 to 56.61	0.2502	0.02	-0.004143 to 0.03899	0.1099	-30.18	-207.7 to 147.3	0.7320
EOTAXIN/CCL11	-55.7	-156.5 to 45.13	0.2713	0.02	-0.002567 to 0.03407	0.0900	-113.80	-257.8 to 30.26	0.1184
RANTES/CCL5	-68.6	-181.4 to 44.23	0.2266	0.004	-0.01666 to 0.02434	0.7071	-123.90	-285.1 to 37.27	0.1283
IL-8/CXCL8	-7653	-15638 to 331.0	0.0598	0.37	-1.031 to 1.778	0.5936	-6509.00	-16925 to 3906	0.2133
Growth factors									
G-CSF	356.1	-424.0 to 1136	0.3620	0.10	-0.03098 to 0.2291	0.1317	-733.60	-1766 to 298.7	0.1588
GM-CSF	-20.19	-35.16 to -5.229	**0.0096**	0.003	-0.0000714to 0.005429	0.0559	-3.11	-25.11 to 18.88	0.7755
VEGF	5955	-20628 to 3389	0.1550	1.05	-1.312 to 2.941	0.4442	8625.00	-27903 to 6886	0.2297
FGF basic	-63.56	-154.9 to 27.80	0.1680	0.01	-0.005870 to 0.02664	0.2048	-93.14	-223.5 to 37.19	0.1570
PDFG-BB	-1351	-4402 to 1700	0.3754	0.35	-0.1875 to 0.8833	0.1961	-5402.00	-9444 to -1360	**0.0102**

Bold indicates statistically significant results.

### ROC analysis

**Figure 3:**
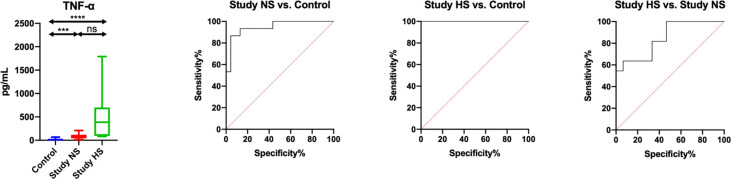
Diagnostic significance of salivary TNF-α for evaluating salivary gland inflammation in patients heart failure and hyposalivation (HS) compared to patients with heart failure (n = 13) and normal saliva secretion (n = 17) (NS) and to the control (n = 30). ***p< 0.001, ****p< 0.0001, ns, non significance.

ROC analysis is a useful method for assessing the predictive accuracy of a model by plotting the sensitivity against 1-specificity of a classification test. In our study, we showed that salivary TNF-α, INF-γ, IL-12 and EOTAXIN/CCL11 differentiated patients with HF and hyposalivation with the highest sensitivity and specificity compared to patients with normal salivary secretion and controls. Of particular note is salivary TNF-α, which has the greatest diagnostic utility (study NS vs. control: AUC = 0.95, sensitivity = 86.67, specificity = 86.96; study HS vs. control: AUC = 1, sensitivity = 100, specificity = 100; study HS vs. study NS: AUC = 0.85, sensitivity = 63.64, specificity = 66.67) (**Figure 3**; **Table S4**).

The results are presented in box-and-whisker plots as median (minimum-maximum). Kruskal-Wallis ANOVA analysis of variance along with Dunn’s *post hoc* test were applied for comparisons between the multiple groups. Analysis of the diagnostic utility of biomarkers was measured using the receiver operating characteristic (ROC) analysis.

**Figure 4:**
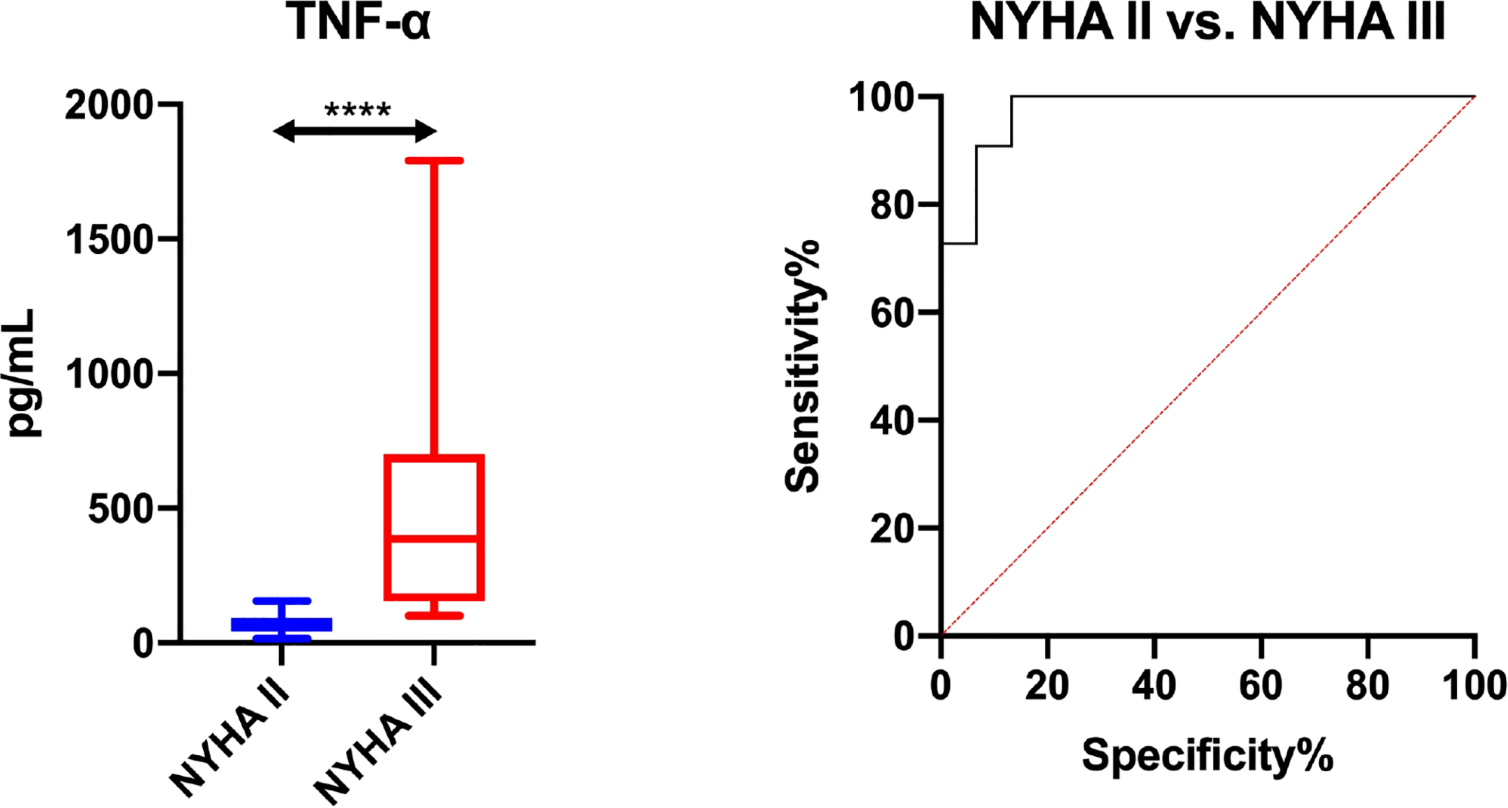
Diagnostic significance of salivary TNF-α for evaluating severity of heart failure symptoms according to the New York Heart Association classification (NYHA II vs. NYHA III). The results are presented in box-and-whisker plots as median (minimum-maximum). Kruskal-Wallis ANOVA analysis of variance along with Dunn’s *post hoc* test was applied for comparisons between the multiple groups. Analysis of the diagnostic utility of biomarkers was measured using the receiver operating characteristic (ROC) analysis. ****p< 0.0001.

The assessed salivary inflammatory and anti-inflammatory biomarkers do not differentiate HF patients by NYHA classes (NYHA III vs. NYHA II), and thus cannot be used in differential diagnosis of heart function (**Table S4**). The exception is salivary TNF-α, which, with an AUC close to unity (AUC = 0.99; sensitivity = 90. 92%, specificity = 93.3%), differentiates HF patients by severity of heart failure symptoms according to the New York Heart Association classification (**Figure 4**; **Table S5**).

## Discussion

The present study is the first to evaluate whether salivary inflammatory and anti-inflammatory factors may be related with the occurrence of hyposalivation in HF patients. We demonstrated significantly higher levels of pro-inflammatory cytokines, anti-inflammatory cytokines, Th1, Th2, Th17, chemokines and growth factors in unstimulated saliva of HF patients compared to controls. Multivariate regression analysis showed that the content of salivary cytokines, chemokines and growth factors is highly dependent on salivary gland function.

Heart failure ranks among the leading causes of death worldwide, which may be due to the multifactorial etiology of the disease. Factors that trigger heart failure (i.e., genetic conditions, infections, atherosclerosis, hypertension, diabetes, alcoholism) as well as processes that have already started in HF patients (e.g., neurohormonal activation, extracellular matrix remodeling, oxidative stress) ultimately lead to increased production and secretion of inflammatory mediators (33, 34). They play a role not only in the initiation but also in the progression of HF (35). Several studies have shown increased expression of cytokines such as TNF-α, IL-1, -6, -18, cardiotrophin-1 (CT-1) and Fas ligand (type II transmembrane protein [homotrimer] which is a cytokine of the TNF family), as well as numerous chemokines (e.g., monocyte chemoattractant (MCP)-1/CCL2, IL-8/CXCL8 and macrophage inflammatory protein-1α/CCL3) in patients with HF (34–36). Interestingly, elevated plasma levels of inflammatory cytokines and chemokines correlate with deterioration in the functional class (the New York Heart Association classification – NYHA) and cardiac function (left ventricular ejection fraction – LVEF). It is not surprising that in patients with HF, the effect of inflammation has been increasingly postulated not only in relation to myocardial and vascular damage, but also to salivary hypofunction. In HF patients, salivary gland dysfunction is manifested as a reduction in SFR, a decrease in salivary protein secretion or a decrease in salivary amylase activity (18, 37). Unfortunately, the causes of salivary hypofunction in patients with HF are still not well understood.

Saliva is mainly produced by three large paired salivary glands (parotid, sublingual and submandibular) and, to a lesser extent, by numerous small glands located in the oral mucosa. Under physiological conditions, the total volume of saliva per day varies from 0.5 to 1 liter in an adult, and about 80% of this volume depends on the stimulation associated with food intake. The parasympathetic nervous system is dominant in saliva secretion, stimulating the secretion of a large volume of watery saliva with a relatively low TP. Acetylcholine acts on M1 and M3 receptors in the acinar cells causing the release of calcium ions from intracellular stores and the opening of Ca^2+^-dependent chloride and potassium channels. Stimulation of the sympathetic nervous system has little effect on saliva volume, with its main role being to modify saliva composition. Norepinephrine acting on α1 receptors stimulates the secretion of watery saliva, and stimulation of β1 receptors results in increased secretion of proteins into saliva (38–40). It should also be noted that salivary glands are very well vascularized, ensuring the efficient passage of many compounds into saliva. Reduced saliva secretion leads to a number of health consequences. Patients with hyposalivation experience symptoms of dry mouth, difficulty speaking and swallowing food and taste perception disorders (with a predominance of bitter and salty taste) or a complete lack of taste sensations. Moreover, hyposalivation leads to mechanical damage and a change in the color of the oral epithelium and is accompanied by an annoying and unpleasant odor from the mouth. In a more advanced stage of the disease, we can observe cases of severe and chronic candidiasis (infection caused by fungi of the genus *Candida*), ulceration of tissues, chronic infections caused by bacteria of the genus *Streptococcus* and *Lactobacillus*, which eventually leads to acute forms of circular caries and the formation of tooth cervical defects (41–43).

This study is the first to evaluate the salivary inflammatory profile in patients with HF. We demonstrated significantly higher levels of pro-inflammatory cytokines (IL-1β, TNF-α, IL-7), anti-inflammatory cytokines (IL-1ra, IL-13), Th1 (INF-γ, IL-12, IL-2, IL-15), Th2 (IL-5, IL-6, IL-9), Th17 (IL-17), chemokines (MCP-1/CCL-2, MIP-1α/CCL3, MIP-1β/CCL4, EOTAXIN/CCL11, RANTES/CCL5, IL-8/CXCL8) and growth factors (GM-CSF, VEGF, FGF basic, PDFG-BB) in unstimulated saliva of HF patients compared to the controls. Interestingly, the content of some pro- and anti-inflammatory mediators in saliva significantly exceeds their plasma levels (control: IL-1β, TNF-α, IL-7, IL-1ra, INF-γ, IL-12, IL-4, IL-6, MCP-1/CCL-2, IL-8/CXCL8, GM-CSF, VEGF; HF: IL-1β, TNF-α, IL-7, IL-10, IL-1RA, IL-13, INF-γ, IL-12, IL-4, IL-9, IP-10/CXCL10, MCP-1/CCL-2, IL-8/CXCL8, GM-CSF, VEGF, FGF basic, PDFG-BB), which may confirm previous reports on the production of cytokines, chemokines and growth factors within the salivary glands (25, 44, 45). Indeed, compounds found in saliva can be transported into the oral cavity from plasma *via* intracellular (passive or specific transport) or extracellular (diffusion or ultrafiltration) routes. They can also be synthesized in the salivary glands (mainly parotid and submandibular) (46). It is well known that most cytokines are not stored but produced in cells and released in response to a stimulus. In our previous studies, we confirmed the key contribution of oxidative stress to salivary-gland hypofunction in HF patients (17, 18). These patients demonstrate the accumulation of oxidation/glycation products in the parenchyma of the salivary glands, which may lead to morphological changes in glandular tissue, thus impairing saliva secretion and disrupting salivary protein biosynthesis. A significant role in this process may be played by AGEs which, by binding to a specific receptor (RAGE), not only enhance the production of free radicals, but also activate the pro-inflammatory cascade (19, 20, 47). However, it is interesting to note the lack of correlation between salivary and plasma concentrations of the biomarkers assessed in patients with HF. This may suggest a different nature/severity of inflammatory changes at the central (blood) and local (salivary glands) level. In contrast, in the control group, the content of assessed biomarkers generally correlated positively with their plasma levels (IL-1β, TNF-α, IL-10, IL-1ra, IL-4, IL-5, IL-6, IL-9, MIP-1β/CCL4, RANTES/CCL5, IL-8/CXCL8, G-CSF, VEGF). This confirms previous reports on the use of saliva to assess the systemic inflammatory profile. Collecting saliva as a diagnostic material does not require incurring high costs, and is non-invasive, painless and convenient for young children as well as the elderly and patients still active on the labor market (46). However, ROC analysis did not confirm the diagnostic utility of salivary cytokines and chemokines in the differential diagnosis of HF patients. The assessed salivary biomarkers do not indicate the severity of heart failure (NYHA III vs. NYHA II). Indeed, previous studies and the present one point to the limited clinical utility of salivary biomarkers in patients with systemic diseases and concomitant salivary gland hypofunction (25, 48).

Hyposalivation belongs to quantitative disorders of saliva secretion. It can be objectively diagnosed *via* measuring the sialometry when the SFR falls below 0.2 ml/min (24–26). Therefore, we examined how the salivary inflammatory profile changes in HF patients with hyposalivation compared to HF patients with normal salivary secretion and the controls. We showed that the levels of selected biomarkers are significantly higher in HF patients with salivary gland dysfunction compared to those with normosalivation and healthy subjects (IL-1β, TNF-α, IL-7, IL-13, INF-γ, IL-12, IL-15, IL-5, IL-6, IL-9, IL-17, MCP-1/CCL-2, EOTAXIN/CCL11, RANTES/CCL5, GM-CSF, VEGF, FGF basic, PDFG-BB). Increased levels of pro-inflammatory cytokines, anti-inflammatory cytokines, Th1, Th2, Th17, chemokines and growth factors in HF patients with hyposalivation may indicate a multidirectional inflammatory process within the salivary glands. However, the obtained results do not indicate a dominance of either branch of the immune response. Since hyposalivation is also accompanied by impaired salivary protein biosynthesis, we investigated the relationship between the saliva inflammatory profile, total protein content and activity of salivary amylase, the main salivary protein which is also the most important biomarker of the salivary-gland secretory dysfunction. Salivary concentrations of IL-1β and TNF-α were negatively correlated with salivary minute flow, protein content and salivary amylase activity in subjects from the study group; no such correlations were found in the control group. The multivariate linear regression analysis demonstrated that the content of salivary cytokines, chemokines and growth factors significantly depends on the secretory activity of the salivary glands. Salivary IL-1β and TNF-α concentrations depend inversely on SFR, TP and SA, INF-γ and IL-15 – on SFR and SA, and IL-12 levels – on SFR. The exclusion of Sjögren’s syndrome, other systemic diseases and oral inflammation may suggest that the abnormalities observed by us are due to salivary gland dysfunction in the course of HF. Hyposalivation significantly disrupts the homeostasis of the oral ecosystem. Lower concentrations of salivary defense proteins (immunoglobulins) increase the risk of fungal and bacterial infections, while limited dilution of sugars and removal of food from the tooth surface contribute to a significant increase in the risk of caries and periodontitis (49, 50). Hyposalivation also predisposes the oral cavity to more frequent mechanical damage, which may result in the development of erosions and premalignant and/or neoplastic conditions of the oral cavity (51, 52). Using ROC analysis, we showed that salivary TNF-α, INF-γ, IL-12 and EOTAXIN/CCL11 differentiated patients with HF and hyposalivation with the highest sensitivity and specificity compared to HF patients with normal salivary secretion and controls. However, salivary TNF-α appears to have the greatest diagnostic utility for assessing the degree of salivary gland failure in patients with HF. This biomarker is the most dependent on SFR, salivary protein content and amylase activity, and its salivary concentration does not correlate with blood levels.

The salivary inflammatory profile has not been evaluated before in patients with HF. Similarly, little is known about the role of inflammation in disrupting the salivary gland function in the course of systemic diseases. However, according to previous studies, INF-γ decreases the production of mucins in acinar cells of patients with Sjögren’s syndrome (53). Mucins belong to a group of the most important oral proteins. In unstimulated saliva, they account for as much as 20–30% of total content of proteins. These compounds determine the rheological properties of saliva, enabling it to coat and moisten the oral tissues and reduce frictional forces. They also participate in the processes of agglutination and colonization of bacteria. In addition, INF-γ can enhance salivary gland remodeling by increasing the activity of matrix metalloproteinases (MMPs) (54). These belong to a group of proteolytic enzymes that digest components of the extracellular matrix and basement membrane of the salivary glands. Thus, MMPs disrupt salivary-gland secretory function. Similar to TNF-α, IL-6 and IL-12, INF-γ also activates intrinsic apoptosis pathways (55–57). It has been suggested that overproduction of salivary INF-γ, IL-1α, IL-1β, IL-1ra and IL-12 may originate from inflammatory cells that infiltrate the salivary glands. It is well known that these cytokines are released by stimulated monocytes, macrophages and vascular endothelial cells. Indeed, the site of inflammation is a place of accumulation of numerous immunocompetent cells necessary for a full immune response to occur. The activated antigen-presenting cell secretes pro-inflammatory cytokines, the anti-inflammatory interleukin IL-10, and chemokines. The presenting cells have receptors of these molecules on their surface, which autocrinically increases their activation. Elevated levels of salivary IL-6 have been shown to correlate significantly with the degree of lymphocytic infiltration in the labial salivary glands of patients with Sjögren’s syndrome (58, 59). IL-6 significantly boosts the local inflammatory process, inducing T-lymphocyte proliferation and B-lymphocyte differentiation, and decreasing the number of Treg lymphocytes (60, 61). In addition, increased secretion and concentration of INF-γ, IL-1 and TNF-α in the inflammatory microenvironment of the salivary glands can inhibit acetylcholine release, resulting in attenuated acinar cell responses and decreased saliva secretion (62).

**Figure 5:**
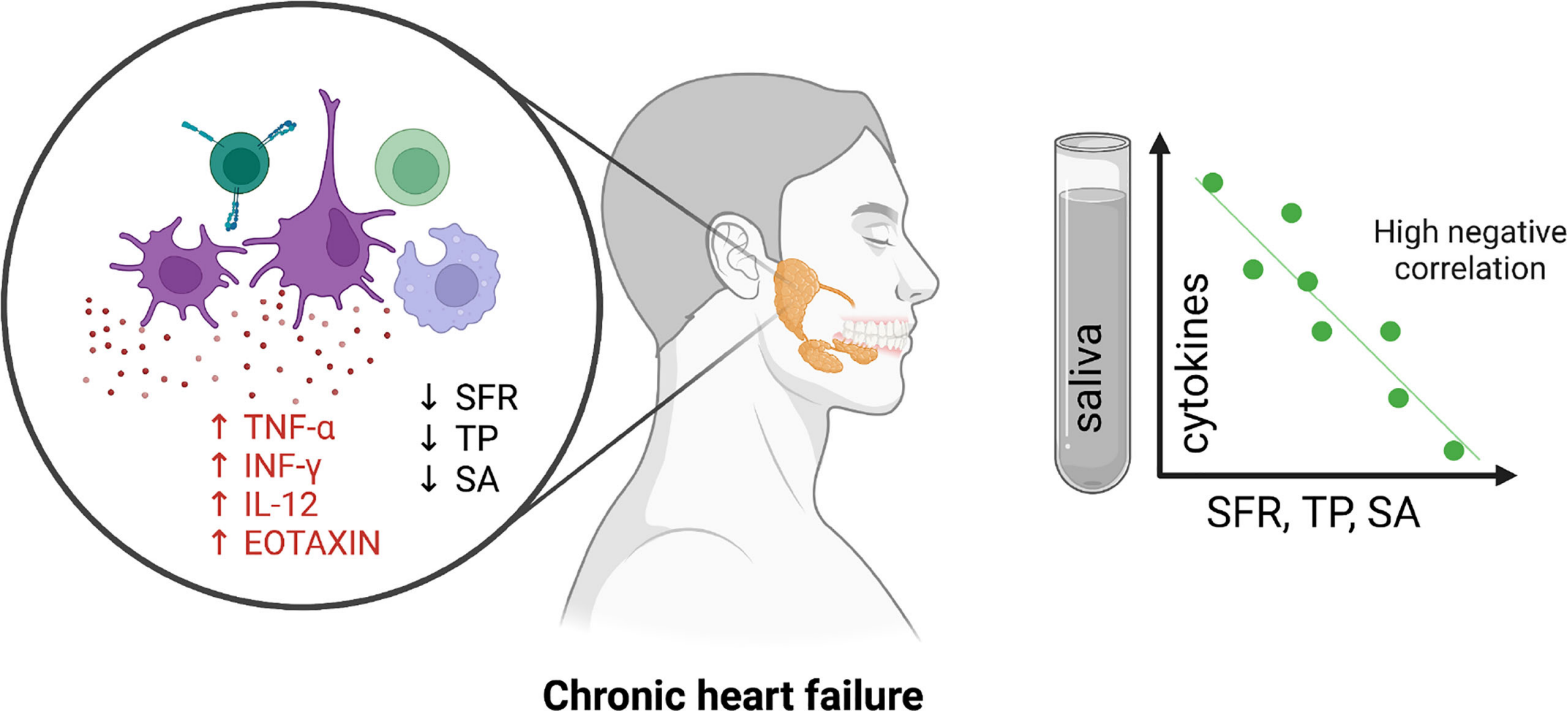
Graphical conclusions from the study.

In conclusion, in HF patients we can observe an abnormal salivary inflammatory profile (**Figure 5**). Although our study was purely observational, the considerably higher concentration of inflammatory parameters in saliva compared to plasma, as well as the lack of saliva-blood correlation, may suggest enhanced production/secretion of these compounds in salivary gland cells of HF patients. Salivary TNF-α, INF-γ, IL-12 and EOTAXIN/CCL11 can be helpful in assessing the progression of inflammation in the salivary glands of HF patients. However, ROC analysis did not confirm the diagnostic utility of salivary cytokines and chemokines in the differential diagnosis of HF patients (NYHA III vs. NYHA II). Thus, our study demonstrates the limited clinical utility of salivary biomarkers in HF patients with concomitant salivary gland hypofunction. The exception is salivary TNF-α, which, with an AUC close to unity, differentiates HF patients by severity of heart failure symptoms according to the New York Heart Association classification. However, these results should be interpreted with great caution due to the small number of patients with HF. Further studies are needed to evaluate inflammatory changes at the molecular level in the salivary glands of patients with HF.

A limitation of our study is the low number of patients with HF. However, we would like to underline that according to the sample size calculation such a number of subjects is sufficient for performing an analysis. It is also noteworthy that this is a group of patients selected in a very particular manner: they had healthy periodontium, no oral inflammatory diseases, and no other general diseases (including autoimmune ones). This is of crucial importance, since saliva composition depends on the local condition of the oral cavity and the patient’s overall health status. Therefore, in order to reliably assess salivary gland function in the course of HF, researchers should select patients very rigorously, although the consequence of which may be a small group size. We also realize that saliva secretion is strongly influenced by cardiovascular drugs (63). However, patients in the study group and controls were treated with similar medications to limit the effect of pharmacotherapy on the quantitative and qualitative composition of saliva. In addition, due to ethical constraints, we were not able to collect salivary glands for analysis and only evaluated changes of circulating inflammatory biomarkers. Nevertheless, we have analyzed them closely with biological functions (i.e., secretory activity of salivary glands).

## Data availability statement

The original contributions presented in the study are included in the article/**Supplementary Material**. Further inquiries can be directed to the corresponding author.

## Ethics statement

The studies involving human participants were reviewed and approved by Bioethics Committee of the Medical University of Bialystok, Poland (permission number R-I-002/75/2016). The patients/participants provided their written informed consent to participate in this study.

## Author contributions

Conceptualization, AK, AZ, and MM; Data curation, AK and MM; Formal analysis, AK and MM; Funding acquisition, AZ and MM; Investigation, AK, AZ, and MM; Methodology, AK, AZ, and MM; Project administration, AZ and MM; Resources, AK, MK, and AS-R; Software, AS-R; Supervision, AZ and MM; Validation, AK and MM; Visualization, AK and MM; Writing—original draft, AK and MM; Writing—review and editing, AZ, MK, and MM. All authors contributed to the article and approved the submitted version.

## Funding

This work was supported by the Medical University of Bialystok, Poland (Grant No. SUB/1/DN/22/002/3330).

## Conflict of interest

The authors declare that the research was conducted in the absence of any commercial or financial relationships that could be construed as a potential conflict of interest.

## Publisher’s note

All claims expressed in this article are solely those of the authors and do not necessarily represent those of their affiliated organizations, or those of the publisher, the editors and the reviewers. Any product that may be evaluated in this article, or claim that may be made by its manufacturer, is not guaranteed or endorsed by the publisher.

## Glossary

**Table:**

AST	aspartate aminotransferase
BMI	body mass index
CRP	c-reactive protein
DBP	diastolic blood pressure
DMFT	decayed, missing, filled teeth index
EGF	vascular endothelial growth factor
Eotaxin/CCL11	Eotaxin/chemokine ligand 11
FGF basic	fibroblast growth factor
G-CSF	granulocyte colony-stimulating factor
GFR	glomerular filtration rate
GI	gingival index
GM-CSF	granulocyte-macrophage colony-stimulating factor
HCT	hematocrit
HGB	hemoglobin; concentration
HS	hyposalivation
IL-1b	interleukin 1b
IL-1ra	interleukin-1RA
IL-2	interleukin 2
IL-4	interleukin 4
IL-5	interleukin 5
IL-6	interleukin 6
IL-7	interleukin 7
IL-8/CXCL8	interleukin 8
IL-9	interleukin 9
IL-10	interleukin 10
IL-12	interleukin 12
IL-13	interleukin 13
INF-g	interferon g
IL-15	interleukin 15
IL-17	interleukin 17
IP-10/CXCL10	interferon gamma-induced protein 10/chemokine (C-X-C motif) ligand 10
LVEF	left ventricle ejection fraction
MCH	mean corpuscular hemoglobin
MCP-1/CCL2	monocyte chemoattractant protein-1
MIP-1a/CCL3	macrophage inflammatory protein-1 a/chemokine ligands 3
MIP-1b/CCL4	macrophage inflammatory protein-1/b chemokine ligands 4
NS	normal salivation
PBI	papilla bleeding index
PDFG-BB	plateletderived growth factor–BB
PLT	platelets
RBC	red blood cells
RANTES/CCL5	regulated on activation, normal T cell expressed and secreted/chemokine ligand 5
SBP	systolic blood pressure
SFR	saliva flow rate
SA	salivary amylase
TNF-a	tumor necrosis factor a
TSH	thyroid stimulating hormone
WBC	white blood cells
VEGF	vascular endothelial growth factor
