# Supplementary material for: Could inflammation contribute to salivary gland dysfunction in patients with chronic heart failure?
Source: Front Immunol. 2022 Oct 10;13:1005981. doi: 10.3389/fimmu.2022.1005981 (PMC9589450; doi:10.3389/fimmu.2022.1005981)
Supplement: Supplementary file 1 [file Table_1.docx]

**Table S1: Salivary pro-inflammatory and anti-inflammatory profile in patients with heart failure compared to the control**. IL-1β: interleukin 1β; TNF-α: tumor necrosis factor α ; IL-7: interleukin 7; IL-10: interleukin 10; IL-1RA: interleukin 1RA; IL-13: interleukin 13; INF-γ: interferon γ; IL-12: interleukin 12; IL-2: interleukin 2; IL-15: interleukin 15; IL-4: interleukin 4; IL-5: interleukin 5; IL-6: interleukin 6; IL-9: interleukin 9; IL-17: interleukin 17; IP-10/CXCL10: chemokine (C-X-C motif) ligand 10/interferon gamma-induced protein 10; MCP-1/CCL2: monocyte chemoattractant protein-1; MIP-1α/CCL3: chemokine ligands 3/macrophage inflammatory protein-1 α; MIP-1β/CCL4: chemokine ligands 4/macrophage inflammatory protein-1 β; CCL11/Eotaxin: chemokine ligand 11/Eotaxin; CCL5/RANTES: chemokine ligand 5/regulated on activation, normal T cell expressed and secreted; IL-8/CXCL8: interleukin 8; G-CSF: granulocyte colony-stimulating factor; GM-CSF: granulocyte-macrophage colony-stimulating factor; VEGF: vascular endothelial growth factor; FGF basic: fibroblast growth factor; PDFG-BB: platelet-derived growth factor–BB; ND: not detectable.

| **Biomarker** | **Saliva** | | | | | | | | **Plasma** | | | | | | | | **Correlation saliva & plasma** | | | |
| --- | --- | --- | --- | --- | --- | --- | --- | --- | --- | --- | --- | --- | --- | --- | --- | --- | --- | --- | --- | --- |
|  | Control group | | | Study group | | | P-value | Change | Control group | | | Study group | | | P-value | C  h  a  n  g  e |  |  |  |  |
|  |  |  |  |  |  |  |  |  |  |  |  |  |  |  |  |  | Control group | | Study group | |
|  | Median | 25% Percentile | 75% Percentile | Median | 25% Percentile | 75% Percentile |  |  | Median | 25% Percentile | 75% Percentile | Median | 25% Percentile | 75% Percentile |  |  | R | P-value | R | P-value |
| Pro-inflammatory cytokines | | | | | | | | | | | | | | | |  |  |  |  |  |
| IL-1β | 45.26 | 19.3 | 74.2 | 131 | 67.26 | 390 | **0.0002** | ­**↑** | 18.55 | 7.79 | 32.89 | 23.1 | 12.13 | 29.86 | 0.6416 | **≈** | 0.404 | **0.033** | 0.753 | **<0.0001** |
| TNF-α | 13.93 | 9.31 | 21.36 | 98.24 | 51.2 | 254.2 | **<0.0001** | **↑** | 6.99 | 3.11 | 18.14 | 16.94 | 11.61 | 37.5 | **0.0003** | ­**↑** | 0.604 | **0.00614** | 0.347 | 0.052 |
| IL-7 | 16.75 | 11.32 | 21.34 | 41.27 | 32.14 | 53.46 | **<0.0001** | **↑** | 6.93 | 3.9 | 28.85 | 6.14 | 2.42 | 7.28 | 0.1292 | **≈** | 0.29 | 0.12 | 0.049 | 0.869 |
| Anti-inflammatory cytokines | | | | | | | | | | | | | | | |  |  |  |  |  |
| IL-10 | 19 | 11 | 25 | 19.7 | 13.76 | 29.33 | 0.1512 | **≈** | 34.95 | 28.79 | 40.13 | 8.75 | 3.72 | 12.31 | **<0.0001** | **↓** | 0.189 | **0.001** | 0.403 | 0.137 |
| IL-1RA | 4726 | 1904 | 6576 | 6628 | 4487 | 10253 | **0.0081** | **↑** | 32.41 | 18.14 | 437.5 | 18.01 | 10.98 | 268 | 0.2033 | **≈** | 0.557 | **0.001** | 0.34 | 0.143 |
| IL-13 | 0.83 | 0.45 | 1.73 | 2.14 | 1.68 | 2.313 | **<0.0001** | **↑** | 1.44 | 0.65 | 11 | 6 | 1.91 | 10 | 0.2184 | **≈** | 0.018 | 0.928 | -0.146 | 0.505 |
| Th1 cytokines | | | | | | | | | | | | | | | |  |  |  |  |  |
| INF-γ | 19.71 | 10.97 | 28.22 | 85.1 | 62.25 | 106.3 | **<0.0001** | **↑** | 1.41 | 1.03 | 1.86 | 3.07 | 2.23 | 5.67 | **0.0003** | **↑** | 0.401 | 0.088 | -0.592 | **0.001** |
| IL-12 | 32.5 | 20.13 | 31.38 | 39.99 | 36.12 | 54.78 | **<0.0001** | **↑** | 15.29 | 9.52 | 19.68 | 33.81 | 30.73 | 36.12 | **<0.0001** | **↑** | 0.245 | 0.361 | -0.026 | 0.9665 |
| IL-2 | 30.00 | 18.5 | 34.24 | 43.77 | 24.1 | 66.23 | **0.007** | **↑** | ND | ND | ND | ND | ND | ND | ND | ND | ND | ND | ND | ND |
| IL-15 | 102 | 65.66 | 134.5 | 643.2 | 416.4 | 769.1 | **<0.0001** | **↑** | ND | ND | ND | ND | ND | ND | ND | ND | ND | ND | ND | ND |
| Th2 cytokines | | | | | | | | | | | | | | | |  |  |  |  |  |
| IL-4 | 13.5 | 1.133 | 18.5 | 3.285 | 2.113 | 5.09 | **0.0202** | **↓** | 2.33 | 1.64 | 2.83 | 0.24 | 0.1 | 0.43 | **<0.0001** | **↓** | 0.516 | **0.0049** | 0.147 | 0.547 |
| IL-5 | 6.55 | 5 | 8.75 | 133.3 | 81.44 | 195.5 | **<0.0001** | **↑** | 41.14 | 17.91 | 80.27 | 158.5 | 114.1 | 209.2 | **<0.0001** | **↑** | -0.763 | **0.0000175** | 0.5 | **0.018** |
| IL-6 | 5.48 | 4.33 | 7.36 | 16.16 | 8.77 | 42.66 | **<0.0001** | **↑** | 2.32 | 1.245 | 5.47 | 94.43 | 50.29 | 132.6 | **<0.0001** | **↑** | 0.948 | **<0.0001** | -0.021 | 0.929 |
| IL-9 | 44 | 17.9 | 63 | 101.4 | 57.57 | 176.6 | **<0.0001** | **↑** | 73.9 | 33.22 | 146.4 | 35.95 | 20.41 | 59.36 | **0.0022** | **↓** | 0.431 | **0.019** | 0.065 | 0.7575 |
| Th17 cytokines | | | | | | | | | | | | | | | |  |  |  |  |  |
| IL-17 | 5.25 | 4.03 | 10.49 | 36.63 | 21.11 | 54.85 | **<0.0001** | ­ | ND | ND | ND | ND | ND | ND | ND | ND | ND | ND | ND | ND |
| Chemokines | | | | | | | | | | | | | | | |  |  |  |  |  |
| IP-10/CXCL10 | 110.1 | 68.88 | 270.1 | 121.3 | 37.73 | 1622 | 0.8949 | **≈** | 233 | 143.2 | 351.8 | 99.12 | 68.07 | 135.6 | **0.0015** | **↓** | -0.169 | 0.563 | 0.256 | 0.338 |
| MCP-1/CCL-2 | 63.57 | 28.63 | 133 | 908.6 | 437 | 1436 | **<0.0001** | **↑** | 21.98 | 17.13 | 24.66 | 8.45 | 5.36 | 15.03 | **0.0003** | **↓** | -0.154 | 0.6153 | 0.426 | 0.114 |
| MIP-1α/CCL3 | 1.23 | 0.84 | 3.88 | 2.43 | 1.97 | 3.58 | **0.0209** | **↑** | 1.97 | 1.34 | 13.32 | 4.7 | 0.59 | 13.89 | 0.4851 | **≈** | 0.205 | 0.348 | -0.172 | 0.48 |
| MIP-1β/CCL4 | 25.33 | 5.35 | 44.94 | 33.64 | 21.22 | 69.19 | **0.0238** | **↑** | 40.54 | 18.88 | 109.3 | 112.5 | 64.28 | 168.3 | **0.0022** | ­**↑** | 0.637 | **0.006** | -0.26 | 0.256 |
| EOTAXIN/CCL11 | 20.14 | 15.29 | 25.59 | 52.12 | 36.98 | 65.7 | **<0.0001** | **↑** | 104.7 | 69.8 | 269.9 | 110.7 | 60.29 | 139.5 | 0.2408 | **≈** | 0.128 | 0.559 | -0.069 | 0.745 |
| RANTES/CCL5 | 4.43 | 3 | 8.12 | 25.44 | 22.86 | 33.89 | **<0.0001** | **↑** | 649.4 | 285.2 | 1495 | 1555 | 1125 | 2319 | **0.0016** | ­**↑** | -0.717 | **0.0001184** | 0.1 | 0.666 |
| IL-8/CXCL8 | 1134 | 225.7 | 1575 | 1996 | 1337 | 4227 | **0.0068** | **↑** | 24.91 | 15.48 | 29.53 | 9.24 | 6.137 | 20.69 | **0.0025** | **↓** | 0.645 | **0.0001594** | -0.1 | 0.769 |
| Growth factors | | | | | | | | | | | | | | | |  |  |  |  |  |
| G-CSF | 497 | 413.5 | 518.3 | 358 | 266.6 | 670 | 0.4424 | **≈** | 487.2 | 198.6 | 592.1 | 683.2 | 541.4 | 735 | **0.0061** | **↑** | 0.44 | **0.041** | 0.085 | 0.729 |
| GM-CSF | 2.12 | 1.87 | 2.64 | 6.27 | 4.34 | 8.21 | **<0.0001** | **↑** | 0.9945 | 0.8668 | 1.069 | 2.39 | 1.9 | 3.125 | **<0.0001** | **↑** | 0.113 | 0.666 | 0.352 | 0.319 |
| VEGF | 967.9 | 295.2 | 1795 | 2616 | 1700 | 6855 | **<0.0001** | **↑** | 163.3 | 123.9 | 192.3 | 191.4 | 117.6 | 372.2 | 0.1966 | **≈** | 0.582 | **0.003** | -0.113 | 0.636 |
| FGF basic | 5.99 | 5.22 | 11.3 | 46.65 | 40.86 | 59.08 | **<0.0001** | **↑** | 30.19 | 21.45 | 33.52 | 31.48 | 26.33 | 38.91 | 0.3475 | **≈** | 0.019 | 0.927 | 0.165 | 0.488 |
| PDFG-BB | 490 | 360 | 580 | 2085 | 1806 | 2453 | **<0.0001** | **↑** | ND | ND | ND | ND | ND | ND | ND | ND | ND | ND | ND | ND |

**Table S2. Salivary pro-inflammatory and anti-inflammatory profile in patients with heart failure and hyposalivation compared to patients with heart failure and normal saliva secretion and to the control.** HS: hyposalivation; NS: normal salivation; IL-1β: interleukin 1β; TNF-α: tumor necrosis factor α; IL-7: interleukin 7; IL-10: interleukin 10; IL-1RA: interleukin 1RA; IL-13: interleukin 13; INF-γ: interferon γ; IL-12: interleukin 12; IL-2: interleukin 2; IL-15: interleukin 15; IL-4: interleukin 4; IL-5: interleukin 5; IL-6: interleukin 6; IL-9: interleukin 9; IL-17: interleukin 17; IP-10/CXCL10: chemokine (C-X-C motif) ligand 10/interferon gamma-induced protein 10; MCP-1/CCL2: monocyte chemoattractant protein-1; MIP-1α/CCL3: chemokine ligands 3/macrophage inflammatory protein-1 α; MIP-1β/ CCL4: chemokine ligands 4/macrophage inflammatory protein-1 β; CCL11/Eotaxin: chemokine ligand 11/Eotaxin; CCL5/RANTES: chemokine ligand 5/regulated on activation, normal T cell expressed and secreted; IL-8/CXCL8: interleukin 8; G-CSF: granulocyte colony-stimulating factor; GM-CSF: granulocyte-macrophage colony-stimulating factor; VEGF: vascular endothelial growth factor; FGF basic: fibroblast growth factor; PDFG-BB: platelet-derived growth factor–BB; NS: normal salivation; HS: hyposalivation.

| **Biomarker** | **Control Group** | | | **Study NS** | | | **Study HS** | | | Kruskal Wallis P-value | Control vs. Study NS P-value | Control vs. Study HS P-value | Study NS vs. Study HS P-value |
| --- | --- | --- | --- | --- | --- | --- | --- | --- | --- | --- | --- | --- | --- |
|  | Median | 25% Percentile | 75% Percentile | Median | 25% Percentile | 75% Percentile | Median | 25% Percentile | 75% Percentile |  |  |  |  |
| Pro-inflammatory cytokines | | | | | | | | | | | | | |
| IL-1β | 45.26 | 19.3 | 74.2 | 82.34 | 37.41 | 257.3 | 371.7 | 67.53 | 770.3 | **0.0003** | 0.107 | **0.0003** | 0.2227 |
| TNF-α | 13.93 | 9.31 | 21.36 | 70.18 | 43.18 | 117.5 | 385.8 | 95.77 | 701.4 | **<0.0001** | **0.0002** | **<0.0001** | 0.2112 |
| IL7 | 16.75 | 11.32 | 21.34 | 42.74 | 36 | 61.71 | 35.24 | 25.71 | 46.02 | **<0.0001** | **<0.0001** | **<0.0001** | >0.9999 |
| Anti-inflammatory cytokines | | | | | | | | | | | | | |
| IL-10 | 19 | 11 | 25 | 21.9 | 16.33 | 30.26 | 16.31 | 11.43 | 25.98 | 0.1163 | 0.1358 | >0.9999 | 0.409 |
| IL-1RA | 4726 | 1904 | 6576 | 6628 | 4777 | 13374 | 6774 | 2668 | 9973 | **0.0254** | **0.0235** | 0.7652 | >0.9999 |
| IL-13 | 0.83 | 0.45 | 1.73 | 2.14 | 1.828 | 2.535 | 1.91 | 1.44 | 2.14 | **<0.0001** | **<0.0001** | **0.0129** | >0.9999 |
| Th1 cytokines | | | | | | | | | | | | | |
| INF-γ | 19.71 | 10.97 | 28.22 | 69.41 | 45.5 | 99.29 | 95.38 | 73.15 | 132.8 | **<0.0001** | **0.0001** | **<0.0001** | 0.3929 |
| IL-12 | 32.5 | 20.13 | 31.38 | 34.58 | 29.58 | 44.65 | 52.43 | 39.99 | 61.04 | **<0.0001** | **0.0131** | **<0.0001** | 0.05 |
| IL-2 | 30 | 18.5 | 34.24 | 45.08 | 32.89 | 67.12 | 30.03 | 21 | 62.63 | 0.0138 | 0.0107 | 0.6133 | 0.6797 |
| IL-15 | 102 | 65.66 | 134.5 | 643.2 | 420.9 | 766.9 | 505.8 | 266.5 | 805.7 | **<0.0001** | **<0.0001** | **0.0003** | >0.9999 |
| Th2 cytokines | | | | | | | | | | | | | |
| IL-4 | 13.5 | 1.133 | 18.5 | 3.555 | 2.535 | 5.895 | 2.375 | 1.438 | 3.478 | **0.0324** | 0.5524 | **0.0306** | 0.6545 |
| IL-5 | 6.55 | 5 | 8.75 | 136.9 | 87.75 | 223.7 | 81.44 | 47.8 | 174.1 | **<0.0001** | **<0.0001** | **0.0005** | 0.6665 |
| IL-6 | 5.48 | 4.33 | 7.36 | 12.96 | 7.81 | 42.38 | 16.9 | 9.7 | 56.76 | **<0.0001** | **0.0002** | **0.0004** | >0.9999 |
| IL-9 | 44 | 17.9 | 63 | 119.9 | 62.32 | 194.2 | 84.97 | 42.52 | 162.7 | **<0.0001** | **<0.0001** | **0.0094** | >0.9999 |
| Th17 cytokines | | | | | | | | | | | | | |
| IL-17 | 5.25 | 4.03 | 10.49 | 36.63 | 22.1 | 56.33 | 34.35 | 20.26 | 55.32 | **<0.0001** | **<0.0001** | **0.0002** | >0.9999 |
| Chemokines | | | | | | | | | | | | | |
| IP-10/CXCL10 | 110.1 | 68.88 | 270.1 | 293.1 | 41.34 | 2777 | 45.48 | 27.77 | 843.6 | 0.2017 | >0.9999 | 0.787 | 0.2233 |
| MCP-1/CCL-2 | 63.57 | 28.63 | 133 | 992.8 | 483.7 | 2030 | 908.6 | 387.8 | 1252 | **<0.0001** | **<0.0001** | **0.0013** | >0.9999 |
| MIP-1α/CCL3 | 1.23 | 0.84 | 3.88 | 2.9 | 2.07 | 4.58 | 2.295 | 1.97 | 3.135 | 0.0514 | 0.0513 | 0.5388 | >0.9999 |
| MIP-1β/CCL4 | 25.33 | 5.35 | 44.94 | 32.75 | 20.48 | 70.58 | 33.64 | 21.22 | 63.35 | 0.0773 | 0.1084 | 0.2773 | >0.9999 |
| EOTAXIN/CCL11 | 20.14 | 15.29 | 25.59 | 59.01 | 50.21 | 70.49 | 41.64 | 25.93 | 50.84 | **<0.0001** | **<0.0001** | **0.009** | 0.1836 |
| RANTES/CCL5 | 4.43 | 3 | 8.12 | 29.03 | 23.19 | 39 | 23.19 | 18.18 | 29.85 | **<0.0001** | **<0.0001** | **0.0042** | 0.6419 |
| IL-8/CXCL8 | 1134 | 225.7 | 1575 | 1854 | 1407 | 3517 | 2386 | 1393 | 8053 | **0.0107** | **0.0472** | 0.0684 | >0.9999 |
| Growth factors | | | | | | | | | | | | | |
| G-CSF | 497 | 413.5 | 518.3 | 343.3 | 236.9 | 684.6 | 365.3 | 266.6 | 511.5 | 0.5312 | >0.9999 | 0.7925 | >0.9999 |
| GM-CSF | 2.12 | 1.87 | 2.64 | 6.39 | 4.34 | 8.938 | 4.82 | 3.73 | 6.99 | **<0.0001** | **<0.0001** | **0.0002** | >0.9999 |
| VEGF | 967.9 | 295.2 | 1795 | 2441 | 1747 | 7602 | 3062 | 1592 | 4850 | **0.0002** | **0.0007** | **0.0091** | >0.9999 |
| FGF basic | 5.99 | 5.22 | 11.3 | 52.57 | 45.47 | 59.72 | 44.26 | 31.55 | 54.91 | **<0.0001** | **<0.0001** | **<0.0001** | >0.9999 |
| PDFG-BB | 490 | 360 | 580 | 2178 | 1892 | 2479 | 1851 | 1543 | 2409 | **<0.0001** | **<0.0001** | **0.007** | >0.9999 |

**Table S3: Correlations of salivary biomarkers with secretory activity of the salivary glands. SFR: saliva flow rate; SA: salivary amylase;** TP: total protein; IL-1β: interleukin 1β; TNF-α: tumor necrosis factor α ; IL-7: interleukin 7; IL-10: interleukin 10; IL-1RA: interleukin 1RA; IL-13: interleukin 13; INF-γ: interferon γ; IL-12: interleukin 12; IL-2: interleukin 2; IL-15: interleukin 15; IL-4: interleukin 4; IL-5: interleukin 5; IL-6: interleukin 6; IL-9: interleukin 9; IL-17: interleukin 17; IP-10/CXCL10: chemokine (C-X-C motif) ligand 10/interferon gamma-induced protein 10; MCP-1/CCL2: monocyte chemoattractant protein-1; MIP-1α/CCL3: chemokine ligands 3/macrophage inflammatory protein-1 α; MIP-1β/ CCL4: chemokine ligands 4/macrophage inflammatory protein-1 β; CCL11/Eotaxin: chemokine ligand 11/Eotaxin; CCL5/RANTES: chemokine ligand 5/regulated on activation, normal T cell expressed and secreted; IL-8/CXCL8: interleukin 8; G-CSF: granulocyte colony-stimulating factor; GM-CSF: granulocyte-macrophage colony-stimulating factor; VEGF: vascular endothelial growth factor; FGF basic: fibroblast growth factor; PDFG-BB: platelet-derived growth factor–BB.

| **Biomarker** | **Control group** | | | | | | **Study group** | | | | | |
| --- | --- | --- | --- | --- | --- | --- | --- | --- | --- | --- | --- | --- |
|  | SFR | | TP | | SA | | SFR | | TP | | SA | |
|  | R | P-value | R | P-value | R | P-value | R | P-value | R | P-value | R | P-value |
| Pro-inflammatory cytokines | | | | | | | | | | | | |
| IL-1β | 0.10 | 0.5933 | 0.06 | 0.7587 | 0.05 | 0.7900 | -0.70 | **0.0001** | -0.83 | **<0.0001** | -0.48 | **0.0120** |
| TNF-α | 0.20 | 0.3727 | 0.22 | 0.3116 | -0.04 | 0.8700 | -0.84 | **<0.0001** | -0.80 | **<0.0001** | -0.49 | **0.0110** |
| IL-7 | 0.30 | 0.1103 | 0.36 | 0.0489 | -0.29 | 0.1180 | 0.16 | 0.4620 | 0.26 | 0.2164 | -0.03 | 0.8760 |
| Anti-inflammatory cytokines | | | | | | | | | | | | |
| IL-10 | -0.36 | 0.0779 | 0.08 | 0.7211 | 0.15 | 0.4640 | -0.09 | 0.6790 | 0.19 | 0.3808 | 0.04 | 0.8400 |
| IL-1RA | 0.02 | 0.9173 | 0.14 | 0.4666 | 0.05 | 0.8070 | 0.22 | 0.3470 | 0.05 | 0.8230 | 0.24 | 0.3030 |
| IL-13 | 0.19 | 0.3211 | 0.11 | 0.5703 | -0.36 | 0.0490 | 0.28 | 0.1790 | 0.21 | 0.3232 | -0.19 | 0.3720 |
| Th1 cytokines | | | | | | | | | | | | |
| INF-γ | 0.04 | 0.8565 | 0.13 | 0.5269 | -0.05 | 0.8050 | -0.64 | **0.0003** | -0.63 | **0.0004** | -0.43 | **0.0230** |
| IL-12 | -0.15 | 0.5282 | 0.14 | 0.5600 | -0.55 | 0.0120 | -0.75 | **0.0000** | -0.61 | **0.0006** | -0.59 | **0.0010** |
| IL-2 | 0.03 | 0.8709 | 0.07 | 0.7269 | 0.22 | 0.2830 | -0.03 | 0.9010 | 0.15 | 0.4962 | 0.11 | 0.6080 |
| IL-15 | 0.09 | 0.6581 | 0.16 | 0.4361 | 0.46 | **0.0200** | -0.09 | 0.6860 | -0.08 | 0.7349 | 0.06 | 0.7990 |
| Th2 cytokines | | | | | | | | | | | | |
| IL-4 | 0.05 | 0.8106 | 0.19 | 0.3394 | -0.46 | **0.0130** | 0.30 | 0.1550 | 0.05 | 0.8053 | -0.06 | 0.7980 |
| IL-5 | -0.16 | 0.4456 | -0.07 | 0.7338 | 0.37 | 0.0730 | 0.15 | 0.4880 | 0.35 | 0.0926 | 0.07 | 0.7410 |
| IL-6 | -0.05 | 0.8143 | 0.00 | 0.9953 | 0.07 | 0.7090 | -0.10 | 0.6400 | 0.52 | **0.0112** | -0.07 | 0.7470 |
| IL-9 | 0.06 | 0.7771 | 0.27 | 0.1610 | -0.36 | 0.0540 | 0.16 | 0.4560 | 0.24 | 0.2541 | -0.06 | 0.7950 |
| Th17 cytokines | | | | | | | | | | | | |
| IL-17 | 0.21 | 0.4987 | -0.39 | 0.1932 | 0.02 | 0.9600 | -0.17 | 0.4490 | 0.12 | 0.5910 | 0.08 | 0.7140 |
| Chemokines | | | | | | | | | | | | |
| IP-10/CXCL10 | -0.24 | 0.3820 | 0.08 | 0.7925 | 0.09 | 0.7430 | 0.14 | 0.5140 | -0.03 | 0.8876 | -0.14 | 0.5110 |
| MCP-1/CCL-2 | -0.33 | 0.2084 | 0.18 | 0.5050 | 0.35 | 0.1800 | 0.11 | 0.6160 | 0.23 | 0.2992 | -0.01 | 0.9640 |
| MIP-1α/CCL3 | -0.09 | 0.6844 | 0.04 | 0.8559 | 0.35 | 0.1000 | 0.19 | 0.3970 | 0.14 | 0.5403 | 0.03 | 0.9010 |
| MIP-1β/CCL4 | -0.26 | 0.3080 | 0.07 | 0.7874 | 0.04 | 0.8910 | 0.02 | 0.9210 | 0.18 | 0.4168 | -0.27 | 0.2180 |
| EOTAXIN/CCL11 | -0.16 | 0.4768 | -0.01 | 0.9679 | 0.25 | 0.2480 | 0.26 | 0.2250 | 0.14 | 0.5174 | -0.10 | 0.6360 |
| RANTES/CCL5 | 0.35 | 0.1057 | -0.07 | 0.7519 | -0.11 | 0.6100 | 0.25 | 0.2340 | 0.21 | 0.3324 | -0.06 | 0.7850 |
| IL-8/CXCL8 | -0.05 | 0.8036 | 0.20 | 0.3068 | -0.08 | 0.6880 | -0.08 | 0.8080 | -0.18 | 0.5537 | -0.18 | 0.5660 |
| Growth factors | | | | | | | | | | | | |
| G-CSF | -0.30 | 0.1643 | 0.29 | 0.1748 | 0.01 | 0.9500 | 0.13 | 0.5460 | 0.34 | 0.1123 | -0.13 | 0.5650 |
| GM-CSF | 0.01 | 0.9716 | -0.16 | 0.5417 | -0.03 | 0.9150 | -0.18 | 0.4170 | 0.09 | 0.6928 | 0.16 | 0.4620 |
| VEGF | -0.10 | 0.6304 | 0.29 | 0.1633 | 0.04 | 0.8560 | -0.21 | 0.3250 | 0.09 | 0.6846 | 0.01 | 0.9490 |
| FGF basic | -0.01 | 0.9735 | -0.05 | 0.8176 | 0.52 | **0.0070** | 0.15 | 0.4760 | 0.14 | 0.5276 | -0.04 | 0.8590 |
| PDFG-BB | -0.07 | 0.7657 | -0.05 | 0.8385 | 0.18 | 0.4070 | 0.27 | 0.2740 | 0.10 | 0.6894 | 0.31 | 0.2160 |

**Table S4. Receiver operating characteristic (ROC) analysis of salivary pro-inflammatory and anti-inflammatory profile in patients with heart failure and hyposalivation compared to patients with heart failure and normal saliva secretion and to the control.** IL-1β: interleukin 1β; TNF-α: tumor necrosis factor α ; IL-7: interleukin 7; IL-10: interleukin 10; IL-1RA: interleukin 1RA; IL-13: interleukin 13; INF-γ: interferon γ; IL-12: interleukin 12; IL-2: interleukin 2; IL-15: interleukin 15; IL-4: interleukin 4; IL-5: interleukin 5; IL-6: interleukin 6; IL-9: interleukin 9; IL-17: interleukin 17; IP-10/CXCL10: chemokine (C-X-C motif) ligand 10/interferon gamma-induced protein 10; MCP-1/CCL2: monocyte chemoattractant protein-1; MIP-1α/CCL3: chemokine ligands 3/macrophage inflammatory protein-1 α; MIP-1β/CCL4: chemokine ligands 4/macrophage inflammatory protein-1 β; CCL11/Eotaxin: chemokine ligand 11/Eotaxin; CCL5/RANTES: chemokine ligand 5/regulated on activation, normal T cell expressed and secreted; IL-8/CXCL8: interleukin 8; G-CSF: granulocyte colony-stimulating factor; GM-CSF: granulocyte-macrophage colony-stimulating factor; VEGF: vascular endothelial growth factor; FGF basic: fibroblast growth factor; PDFG-BB: platelet-derived growth factor–BB; HS: hyposalivation; NS: normal salivation.

| **Biomarker** | **Study NS vs. Control** | | | | **Study HS vs. Control** | | | | **Study HS vs. Study NS** | | | |
| --- | --- | --- | --- | --- | --- | --- | --- | --- | --- | --- | --- | --- |
|  | AUC (95% CI) | P-value | Sensitivity% (95% CI) | Specificity% (95% CI) | AUC (95% CI) | P-value | Sensitivity% (95% CI) | Specificity% (95% CI) | AUC (95% CI) | P-value | Sensitivity% (95% CI) | Specificity% (95% CI) |
| Pro-inflammatory cytokines | | | | | | | | | | | | |
| IL-1β | 0.71 (0.54 to 0.88) | **0.0251** | 66.67 (41.71 to 84.82) | 66.67 (48.78 to 80.77) | 0.88 (0.76 to 1) | **0.0002** | 72.73 (43.44 to 90.25) | 73.33 (55.55 to 85.82) | 0.74 (0.54 to 0.95) | 0.0379 | 63.64 (35.38 to 84.83) | 66.67 (41.71 to 84.82) |
| TNF-α | 0.95 (0.88 to 1) | **<0.0001** | 86.67 (62.12 to 97.63) | 86.96 (67.87 to 95.46) | 1 (1 to 1) | **<0.0001** | 100 (74.12to 100) | 100 (85.69 to 100) | 0.85 (0.7 to 1) | **0.0028** | 63.64 (35.38 to 84.83) | 66.67 (41.71% to 84.82) |
| IL7 | 0.96 (0.89 to 1) | **<0.0001** | 93.75 (71.67to 99.6) | 93.33 (78.68 to 98.82) | 0.99 (0.99 to 1) | **<0.0001** | 100 (75.75 to 100) | 96.67 (83.33 to 99.83) | 0.66 (0.45 to 0.87) | 0.1501 | 58.33 (31.95 to 80.67) | 68.75 (44.4 to 85.84) |
| Anti-inflammatory cytokines | | | | | | | | | | | | |
| IL-10 | 0.69 (0.51 to 0.86) | 0.048 | 56.25 (33.18 to 76.9) | 60 (40.74 to 76.6) | 0.52 (0.32 to 0.73) | 0.8203 | 50 (25.38 to 74.62) | 48 (30.03 to 66.5) | 0.67 (0.46 to 0.88) | 0.1314 | 58.33 (31.95to 80.67) | 56.26 (33.18 to 76.9) |
| IL-1RA | 0.74 (0.59 to 0.9) | **0.0081** | 60 (35.75 to 80.18) | 60 (42.32 to 75.41) | 0.65 (0.33 to 0.97) | 0.2518 | 50 (18.76 to 81.24) | 56.67 (39.2 to 72.62) | 0.6 (0.32 to 0.88) | 0.4835 | 50 (18.76 to 81.24) | 53.33 (30.12 to 75.19) |
| IL-13 | 0.85 (0.73 to 0.96) | **0.0001** | 81.25 (56.99 to 93.41) | 80 (62.69 to 90.49) | 0.82 (0.7 to 0.95) | **0.0012** | 58.33 (31.95 to 80.67) | 73.33 (55.55 to 85.82) | 0.67 (0.4623 to 0.88) | 0.1314 | 58.33 (31.95to 80.67) | 68.75 (44.4 to 85.84) |
| Th1 cytokines | | | | | | | | | | | | |
| INF-γ | 0.92 (0.81 to 1) | **<0.0001** | 87.5 (63.98 to 97.78) | 88.46 (71.02 to 96) | 1 (1 to 1 ) | **<0.0001** | 100 (75.75 to 100) | 100 (87.13 to 100) | 0.75 (0.57 to 0.93) | **0.0244** | 66.67 (39.06 to 86.19) | 68.75 (44.4 to 85.84) |
| IL-12 | 0.81 (0.66 to 0.96) | **0.0015** | 75 (50.5 to 89.82) | 70 (48.10 to 85.45) | 1 (1 to 1) | **<0.0001** | 100 (75.75 to 100) | 100 (83.89 to 100) | 0.82 (0.67 to 0.98) | **0.004** | 66.67 (39.06 to 86.19) | 75 (50.5 to 89.82) |
| IL-2 | 0.79 (0.63 to 0.95) | **0.0019** | 68.75 (44.40 to 85.84) | 72 (52.42 to 85.72) | 0.61 (0.37 to 0.85) | 0.311 | 54.55 (28.01 to 78.73) | 56 (37.07 to 73.33) | 0.6 (0.36 to 0.83) | 0.4015 | 54,55 (28.01 to 78.73) | 56.25 (33.18 to 76.9) |
| IL-15 | 1 (1 to 1) | **<0.0001** | 100 (79.61 to 100) | 100 (86.68 to 100) | 0.99 (0.95 to 1) | **<0.0001** | 87.5 (52.91 to 99.36) | 88 (70.04 to 95.83) | 0.6 (0.32 to 0.89) | 0.4197 | 50 (21.52 to 78.48) | 53.33 (30.12 to 75.19) |
| Th2 cytokines | | | | | | | | | | | | |
| IL-4 | 0.66 (0.5 to 0.83) | 0.0729 | 68.75 (44.4 to 85.84) | 67.86 (49.34 to 82.07) | 0.7 (0.53 to 0.86) | **0.0463** | 66.67 (39.06 to 86.19) | 67.86 (49.34 to 82.07) | 0.737 (0.55 to 0.93) | 0.0347 | 66.67 (39.06 to 86.19) | 68.75 (44.4 to 85.84) |
| IL-5 | 1 (1 to 1) | **<0.0001** | 100 (80.64to 100) | 100 (86.68 to 100) | 0.91 (0.76 to 1) | **<0.0001** | 91.67 (64.61to 99.57) | 92 (75.03 to 98.58) | 0.68 (0.47 to 0.89) | 0.1092 | 66.67 (39.06to 86.19) | 68.75 (44.4 to 85.84) |
| IL-6 | 0.87 (0.75 to 0.99) | **<0.0001** | 75 (50.5 to 89.82) | 76.67 (59.07 to 88.21) | 0.88 (0.72 to 1) | **0.0003** | 81.82 (52.3 to 96.77) | 80 (62.69 to 90.49) | 0.56 (0.33 to 0.79) | 0.5873 | 54.55 (28.01 to 78.73) | 56.25 (33.18 to 76.9) |
| IL-9 | 0.9 (0.8 to 1) | **<0.0001** | 75 (50.5 to 89.82) | 75.86 (57.89 to 87.78) | 0.79 (0.63 to 0.96) | **0.0033** | 66.67 (39.06 to 86.19) | 65.52 (47.35 to 80.06) | 0.6 (0.38 to 0.82) | 0.3777 | 58.33 (31.95 to 80.67) | 56.25 (33.18 to 76.9) |
| Th17 cytokines | | | | | | | | | | | | |
| IL-17 | 0.976 (0.92 to 1) | **<0.0001** | 93.75 (71.67 to 99.68) | 92.31 (66.69 to 99.61) | 1 (1 to 1) | **<0.0001** | 100 (72.25 to 100) | 100 (77.19 to 100) | 0.51 (0.27 to 0.74) | 0.958 | 50 (23.66 to 76.34) | 50 (28 to 72) |
| Chemokines | | | | | | | | | | | | |
| IP-10/CXCL10 | 0.58 (0.37 to 0.8) | 0.4408 | 56.25 (33.18 to 76.9) | 53.33 (30.12 to 75.19) | 0.64 (0.4 to 0.88) | 0.2225 | 66.67 (39.06 to 86.19) | 66.67 (41.71 to 84.82) | 0.69 (0.48 to 0.89) | 0.0947 | 58.33 (31.95 to 80.67) | 62.5 (38.64 to 81.52) |
| MCP-1/CCL-2 | 0.96 (0.9050 to 1) | **<0.0001** | 93.75 (71.67 to 99.68) | 93.75 (71.67 to 99.68) | 0.91 (0.76 to 1) | **0.0003** | 90.91 (62.26 to 99.53) | 93.75 (71.67 to 99.68) | 0.59 (0.37 to 0.8) | 0.4592 | 45.45 (21.27 to 71.99) | 50 (28 to 72) |
| MIP-1α/CCL3 | 0.71 (0.54 to 0.88) | **0.0327** | 66.67 (41.71 to 84.82) | 65.22 (44.89 to 81.19) | 0.67 (0.49 to 0.85) | 0.1061 | 58.33 (31.95 to 80.67) | 60.87 (40.79 to 77.84) | 0.64 (0.43 to 0.85) | 0.2319 | 50 (25.38 to 74.62) | 53.33 (30.12 to 75.19) |
| MIP-1β/CCL4 | 0.71 (0.53 to 0.89) | **0.04** | 56.25 (33.18 to 76.9) | 58.82 (36.01 to 78.39) | 0.7 (0.5 to 0.89) | 0.086 | 63.64 (35.38 to 84.83) | 64.71 (41.30 to 82.69) | 0.53 (0.31 to 0.75) | 0.8051 | 45.45 (21.27 to 71.99) | 50 (28 to 72) |
| EOTAXIN/CCL11 | 0,98 (0.95 to 1.00) | **<0.0001** | 93.75 (71.63 to 99.68) | 91.3 (73.2 to98.45) | 0.85 (0.72 to 0.99) | **0.0007** | 75 (46.77 to 91.11) | 73.91 (53.53 to 87.45) | 0.77 (0.58 to o.97) | **0.0148** | 75 (46.77 to 91.11) | 75 (50.50 to 89.82) |
| RANTES/CCL5 | 0.92 (0.8 to 1) | **<0.0001** | 93.75 (71.67 to 99.68) | 91.3 (73.20 to 98.45) | 0.9 (0.79 to 1) | **0.0001** | 91.67 (64.61 to 99.57) | 91.3 (73.20 to 98.45) | 0.744 (0.56 to 0.93) | **0.0309** | 58.33 (31.95 to 80.67) | 62.5 (38.64 to 81.52) |
| IL-8/CXCL8 | 0.79 (0.63 to 0.95) | **0.0095** | 77.78 (45.26 to 96.05) | 79.31 (61.61to 90.15) | 0.75 (0.5 to 1) | **0.0395** | 71.43 (35.89 to 94.92) | 72.41 (54.28 to 85.3) | 0.6 (0.29 to 0.9) | 0.5254 | 57.14 (25.05 to 84.18) | 55.56 (26.67 to 81.12) |
| Growth factors | | | | | | | | | | | | |
| G-CSF | 0.5 (0.28 to 0.72) | 0.9772 | 56.25 (33.18 to 76.90) | 56.52 (36.81 to 74.37) | 0.65 (0.43 to 0.88) | 0.1511 | 72.73 (43.44 to 90.25) | 69.57 (49.13 to 84.40) | 0.54 (0.32 to o.77) | 0.7113 | 45.45 (21.27 to 71.99) | 43.75 (23.1 to 66.82) |
| GM-CSF | 0.97 (0.91 to 1) | **<0.0001** | 93.75 (71.67 to 99.68) | 94.12 (73.02 to 99.70) | 0.95 (0.87 to 1) | <0.0001 | 90.91 (62.26 to 99.53) | 88.24 (65.66 to 97.91) | 0.53 (0.3 to 0.76) | 0.7861 | 54.55 (28.01 to 78.73) | 56.25 (33.18 to 76.90) |
| VEGF | 0.87 (0.76 to 0.97) | **<0.0001** | 75 (50.50 to 89.82) | 75 (55.10 to 88) | 0.78 (0.58 to 0.97) | 0.0073 | 75 (46.77 to 91.11) | 75 (55.10 to 88) | 0.5 (0.27 to 0.73) | >0.9999 | 66.67 (39.06 to 86.19) | 62.5 (38.64 to 81.52) |
| FGF basic | 1 (0.98 to 1) | **<0.0001** | 93.75 (71.67 to 99.68) | 96.15(81.11to 99.80) | 0.98 (0.9387 to 1) | <0.0001 | 91.67 (64.61 to 99.57) | 92.31 (75.86 to 98.63) | 0.68 (0.47 to 0.9 | 0.1042 | 66.67 (39.06 to 86.19) | 56.25 (33.18 to 76.90) |
| PDFG-BB | 1 (1 to 1) | **<0.0001** | 100 (77.19 to 1) | 100 (85.69 to 100) | 1 (1 to 1) | 0.0006 |  |  | 0.69 (0.37 to 1) | 0.2179 | 80 (37.55 to 98.97) | 84.62 (57.77 to 97.27) |

**Table S5. Receiver operating characteristic (ROC) analysis of salivary pro-inflammatory and anti-inflammatory profile in patients with heart failure patients and NYHA II class compared to patients with heart failure and NYHA class III.** IL-1β: interleukin 1β; TNF-α: tumor necrosis factor α ; IL-7: interleukin 7; IL-10: interleukin 10; IL-1RA: interleukin 1RA; IL-13: interleukin 13; INF-γ: interferon γ; IL-12: interleukin 12; IL-2: interleukin 2; IL-15: interleukin 15; IL-4: interleukin 4; IL-5: interleukin 5; IL-6: interleukin 6; IL-9: interleukin 9; IL-17: interleukin 17; IP-10/CXCL10: chemokine (C-X-C motif) ligand 10/interferon gamma-induced protein 10; MCP-1/CCL2: monocyte chemoattractant protein-1; MIP-1α/CCL3: chemokine ligands 3/macrophage inflammatory protein-1 α; MIP-1β/CCL4: chemokine ligands 4/macrophage inflammatory protein-1 β; CCL11/Eotaxin: chemokine ligand 11/Eotaxin; CCL5/RANTES: chemokine ligand 5/regulated on activation, normal T cell expressed and secreted; IL-8/CXCL8: interleukin 8; G-CSF: granulocyte colony-stimulating factor; GM-CSF: granulocyte-macrophage colony-stimulating factor; VEGF: vascular endothelial growth factor; FGF basic: fibroblast growth factor; PDFG-BB: platelet-derived growth factor–BB; HS: hyposalivation; NS: normal salivation.

| **Biomarker** | **NYHA II** | **NYHA III** | AUC (95% CI) | P-value | Sensitivity% (95% CI) | Specificity% (95% CI) |
| --- | --- | --- | --- | --- | --- | --- |
| Pro-inflammatory cytokines | | | | | | |
| IL-1β | 80.33±66.17 | 514.31±234.13 | 0.5 (0.29 to 0.71) | >0.9999 | 53.33 (30.12 to 75.19) | 46.67 (30.12 to 75.19) |
| TNF-α | 69.58±38.3 | 540.35±545.69 | 0.99 (0.93 to 1) | **<0.0001** | 90.91 (62.26 to 99.53) | 93.33 (70.18 to 99.66) |
| IL7 | 51.37±18.6 | 43.73±32.26 | 0.71 (0.44 to 0.98) | 0.1127 | 57.14 (25.05 to 84.18) | 73.33 (48.05 to 89.1) |
| Anti-inflammatory cytokines | | | | | | |
| IL-10 | 24.83±6.85 | 21.29±13.03 | 0.6 (0.29 to 0.9) | 0.4809 | 42.86 (15.82 to 74.95) | 53.33 (30.12 to 75.19) |
| IL-1RA | 7643.74±4005.18 | 8569.17±3067.63 | 0.33 (0.3 to 0.97) | 0.4237 | 75 (30.06 to 98.72) | 73.33 (48.05 to 89.1) |
| IL-13 | 2.485±0.99 | 2.37±2.15 | 0.73 (0.44 to 1) | 0.0907 | 71.43 (35.89 to 94.92 | 80 (54.81 to 92.95) |
| Th1 cytokines | | | | | | |
| INF-γ | 65.34±28.11 | 107.17±39.52 | 0.81 (0.65 to 0.97) | 0.0053 | 76.92 (49.74 to 91.82) | 73.33 (48.05 to 89.1) |
| IL-12 | 32.3±8 | 62.17±25.43 | 0.98 (0.97 to 1) | **<0.0001** | 92.31 (66.69 to 99.61) | 100 (79.61 to 100) |
| IL-2 | 61.59±39.57 | 49.91±47.74 | 0.63 (0.31 to 0.96) | 0.3502 | 50 (18.76 to 81.24) | 60 (35.75 to 80.18) |
| IL-15 | 647.1±252.25 | 653.09±182.83 | 0.58 (0.3 to 0.86) | 0.6106 | 60 (23.07 to 92.89) | 50 (26.80 to 73.2) |
| Th2 cytokines | | | | | | |
| IL-4 | 4.67±2.69 | 3.81±3.75 | 0.68 (0.38 to 0.98) | 0.1922 | 57 (25.05 to 84.18) | 66.67 (41.71 to 84.82) |
| IL-5 | 195.8±132.09 | 112.59±70.3 | 0.72 (0.5 to 0.95) | 0.0976 | 57.14 (25.05 to 84.18) | 60 (35.75 to 80.18) |
| IL-6 | 29.1±21.49 | 18.02±19.78 | 0.67 (0.39 to 0.94) | 0.2429 | 50 (18.76 to 81.24) | 53.33 (30.12 to 75.19) |
| IL-9 | 168.26±127.87 | 84.9±62.72 | 0.79 (0.57 to 1) | **0.0316** | 71.43 (35.89 to 94.92) | 73.33 (48.05 to 89.1) |
| Th17 cytokines | | | | | | |
| IL-17 | 44.26±19.96 | 39.29±31.68 | 0.62 (0.29 to 0.95) | 0.4137 | 50 (18.76 to 81.24) | 60 (35.75 to 80.18) |
| Chemokines | | | | | | |
| IP-10/CXCL10 | 2482.82±4806.96 | 1001±1956.59 | 0.57 (0.3 to 0.84) | 0.5970 | 42.86 (15.82 to 74.95) | 46.67 (24.81 to 69.88) |
| MCP-1/CCL-2 | 1954.08±1981.09 | 401.97±272.62 | 0.9 (0.76 to 1) | **0.0051** | 83.33 (43.65 to 99.15) | 80 (54.81 to 92.95) |
| MIP-1α/CCL3 | 3.36±1.38 | 2.5±1.21 | 0.73 (0.48 to 0.99) | 0.0862 | 57.14 (25.05 to 84.18) | 57.14 (32.59 to 78.62) |
| MIP-1β/CCL4 | 54.7±36 | 33.21±22.57 | 0.67 (0.4 to 0.93) | 0.2429 | 50 (18.76 to 81.24) | 53.33 (30.12 to 75.19) |
| EOTAXIN/CCL11 | 68.67±30.84 | 52.2±30.16 | 0.64 (0.34 to 0.94) | 0.2903 | 57.14 (25.05 to 84.18) | 60 (35.75 to 80.18) |
| RANTES/CCL5 | 34.32±18.41 | 26.73±14.12 | 0.68 (0.41 to 0.95) | 0.1805 | 57.14 (25.05 to 84.18 | 53.33 (30.12 to 75.19) |
| IL-8/CXCL8 | 3922.01±4112.31 | 2973.79±1714.08 | 0.5 (0.14 to 0.86) | >0.9999 | 50 (8.88 to 91.12) | 57.14 (25.05 to 84.18) |
| Growth factors | | | | | | |
| G-CSF | 520.55±293.69 | 355.25±226.13 | 0.69 (0.4 to 0.98) | 0.1857 | 50 (18.76 to 81.24) | 53.33 (30.12 to 75.19) |
| GM-CSF | 7.87±4.76 | 6.58±3.97 | 0.6 (0.28 to 0.92) | 0.4835 | 50 (18.76 to 81.24) | 46.67 (24.81 to 69.88) |
| VEGF | 4655.41±3621.07 | 5315.37±6036.94 | 0.5 (0.21 to 0.8) | 0.9719 | 57.14 (25.05 to 84.18) | 53.33 (30.12 to 75.19) |
| FGF basic | 60.03±20.11 | 48.31±23.71 | 0.72 (0.43 to 1) | 0.1050 | 71.43 (35.89 to 94.92) | 66.67 (41.71 to 84.82) |
| PDFG-BB | 2131.76±566.58 | 1633.89±306.81 | 0.82 (0.62 to 1) | 0.1567 | 100 (17.77 to 100) | 73.33 (48.05 to 89.1) |
